# Nano-Modified Titanium Implant Materials: A Way Toward Improved Antibacterial Properties

**DOI:** 10.3389/fbioe.2020.576969

**Published:** 2020-11-23

**Authors:** Jianqiao Liu, Jia Liu, Shokouh Attarilar, Chong Wang, Maryam Tamaddon, Chengliang Yang, Kegong Xie, Jinguang Yao, Liqiang Wang, Chaozong Liu, Yujin Tang

**Affiliations:** ^1^Department of Orthopaedics, Affiliated Hospital of Youjiang Medical University for Nationalities, Baise, China; ^2^Youjiang Medical University for Nationalities, Baise, China; ^3^Department of Pediatric Orthopaedics, Xin Hua Hospital Affiliated to Shanghai Jiao Tong University School of Medicine, Shanghai, China; ^4^State Key Laboratory of Metal Matrix Composites, School of Materials Science and Engineering, Shanghai Jiao Tong University, Shanghai, China; ^5^College of Mechanical Engineering, Dongguan University of Technology, Dongguan, China; ^6^Institute of Orthopaedic and Musculoskeletal Science, Division of Surgery & Orthopaedic Science, University College London, The Royal National National Orthopaedic Hospital, Stanmore, United Kingdom

**Keywords:** bactericidal, nanostructure, antibacterial, nanoparticles, titanium-implants

## Abstract

Titanium and its alloys have superb biocompatibility, low elastic modulus, and favorable corrosion resistance. These exceptional properties lead to its wide use as a medical implant material. Titanium itself does not have antibacterial properties, so bacteria can gather and adhere to its surface resulting in infection issues. The infection is among the main reasons for implant failure in orthopedic surgeries. Nano-modification, as one of the good options, has the potential to induce different degrees of antibacterial effect on the surface of implant materials. At the same time, the nano-modification procedure and the produced nanostructures should not adversely affect the osteogenic activity, and it should simultaneously lead to favorable antibacterial properties on the surface of the implant. This article scrutinizes and deals with the surface nano-modification of titanium implant materials from three aspects: nanostructures formation procedures, nanomaterials loading, and nano-morphology. In this regard, the research progress on the antibacterial properties of various surface nano-modification of titanium implant materials and the related procedures are introduced, and the new trends will be discussed in order to improve the related materials and methods.

## Graphical Abstract

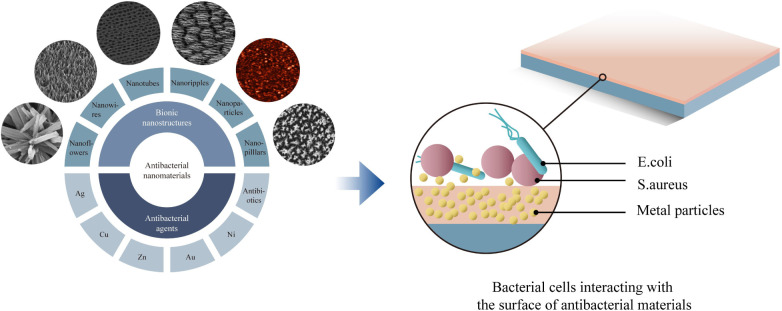

## Introduction

Currently, with the rapid development of materials science and biotechnology, titanium and its alloys as orthopedic implant materials (Sam Froes, 2018) have been widely used in applications such as skeleton structure fixation and joint function repair implants (Gode et al., 2015; Liang et al., 2016; Kaur and Singh, 2019). Many new types of titanium alloys with high-quality performance have been invented through in-depth research on titanium alloy preparation (Zhang C. et al., 2017; Wang et al., 2018; Attarilar et al., 2019a, b; Hafeez et al., 2019) and optimization of titanium alloy composition (Liu et al., 2015a; Wang et al., 2016; Rabadia et al., 2019a, b). These new titanium alloys show outstanding application value in mechanical properties (Guo et al., 2013; Wang et al., 2015; Jawed et al., 2019; Hafeez et al., 2020), corrosion resistance (Lee et al., 2015; Zhu W.Q. et al., 2019; Malhotra et al., 2020), and osteogenic action (Zhu et al., 2016; Li H.F. et al., 2019; Lei et al., 2020).

It is undeniable that the implant material plays a vital role in orthopedic diseases (Hanawa, 2018; Qian et al., 2020), and its infection risk which is directly related to the material condition cannot be ignored (Montanaro et al., 2011; Kumar and Misra, 2018; Li and Webster, 2018). The infection of the tissue in the periphery of implant material is one of the most severe complications in orthopedic surgery (Pfang et al., 2019). The occurrence of infection not only leads to the failure of the implant and the surgery but also increases the patients’ recovery period and makes an economic burden on both patients and the medical system. The use of antibiotics is a common and effective way to control this issue, but it also has some disadvantages (Klein et al., 2016; Holleyman et al., 2019). Bacterial infection on the surface of the implanted material may eventually form a biofilm and reduce or completely inhibit the beneficial effects of the bactericidal drugs. Besides, the system-administered anti-infection method can also result in a low concentration of drugs in the surgical area due to scars or fibrosis of the surrounding tissues, which affects the antibacterial efficiency (Andersson and Hughes, 2012, 2014). For this reason, the preparation of titanium-based implant materials with antibacterial properties can effectively solve this problem. This infection issue can be solved by adding metallic bactericidal elements or the addition of antibacterial coatings on the surface of implants (Morones-Ramirez et al., 2005; Ben-Knaz Wakshlak et al., 2016; Pareek et al., 2018).

The emergence of nanotechnology has caused significant changes in many fields of science, such as physics, chemistry, materials science, biology, computer science. Compared with the conventical materials (Wang et al., 2008), nanoscale materials have many new and unique properties, including medical, mechanical, chemical, magnetic, optical (Whitesides, 2005; Dyakonov et al., 2017; Semenova et al., 2017). Some of the nanoscale materials have appeared as new antibacterial agents, and current studies have confirmed that antimicrobial nanoparticles (NPs) and nanocarriers that aimed to deliver antibiotics can effectively treat infectious diseases (Huh and Kwon, 2011). Nanoscale materials have higher antibacterial properties compared to traditional antibacterial counterparts due to their high surface area to volume ratio. Therefore, it maintains more active area for biological interactions thus this subject seems to have outstanding research value in biomedical applications (Xia, 2008; Avila et al., 2018). This article summarizes and analyzes the advantages and disadvantages, procedures, antibacterial mechanisms, and possible improvement mechanisms for various nanoscale antibacterial materials, in order to provide a guideline for the modern nano-antibacterial materials with improved design and optimum properties for implant applications.

## Classification of Nanomaterials

**FIGURE 1:**
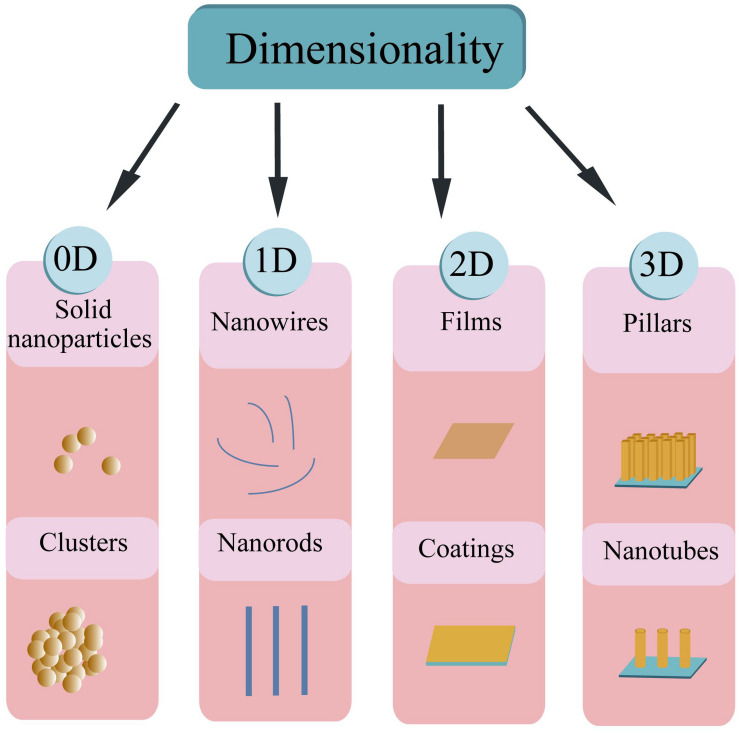
Schematic illustration of the nanomaterial’s classification based on dimensionality.

Based on the dimension criteria, antibacterial nanomaterials can be divided into four categories:zero-dimensional–nanoparticles, one-dimensional–nanowires, two-dimensional—nanofilms, and three-dimensional–nanoblocks (Figure 1) (Saleh, 2020). Besides, antibacterial nanomaterials can also be classified according to the structural form or antibacterial active ingredients (Gleiter, 2000).

### Classification by the Structural Form

Antibacterial nanomaterials based on the structural form can be classified into antibacterial NPs, antibacterial nanosolids, and antibacterial nano-assembled structures. NPs are known as tiny particles in the size range of 1∼100 nm. Their specific structures induce some sort of surface and interface effects such as small size effect, macroscopic quantum tunneling and quantum size effect (Morris, 2018). As a result, nanomaterials with a series of excellent properties enhance the proficiency of nano-antibacterial agents. Compared with ordinary materials, nanomaterials have irreplaceable characteristics, especially in the antibacterial field. Hence it is worthwhile to expand the scope of their applications. Antibacterial nanosolids can be formed by the aggregation of nano-sized antibacterial particles. They can be further divided into bulk, thin-film, and nanofibrous nanomaterials. Antibacterial nano-assembled structures refer to the artificially assembled and synthesized antibacterial nanomaterial systems. These systems are composed of antibacterial nanoparticles, nanofilaments, or tubes as the basic unit. These various nanostructures can be assembled and arranged in one dimensional, two or three-dimensional space to form the desired nanomaterial structures (Mageswari et al., 2016; Khan, 2020).

**FIGURE 2:**
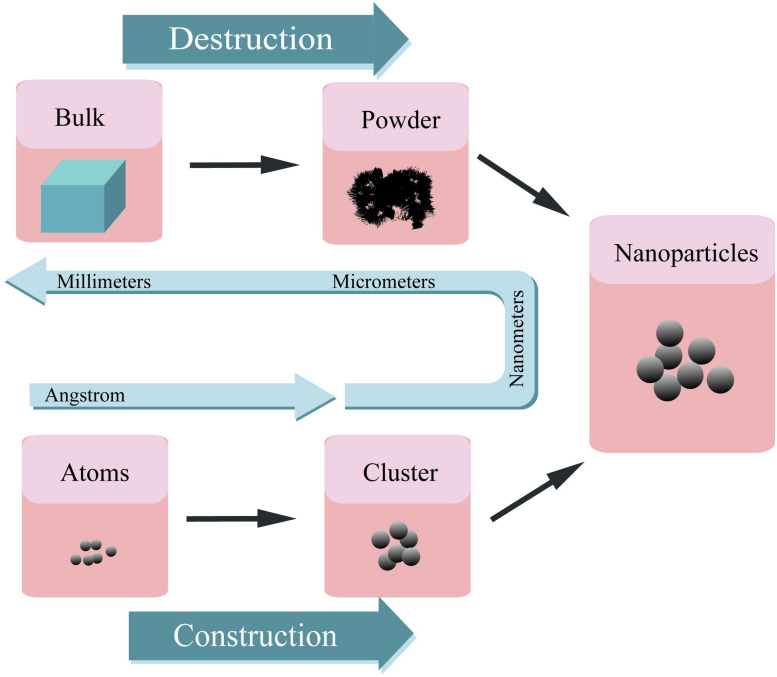
A schematic representation of preparation procedure for various nanoparticles.

Nanomaterials can be synthesized through various methods such as construction and destruction (Figure 2) (Saleh, 2020). On the one hand, NPs can be obtained from the atomic level and then integrated into the desired materials. The methods of this kind of synthesis include self assembly (Zhang et al., 2019b; Deng et al., 2020), laser pyrolysis (Laurent et al., 2010; Dumitrache et al., 2019), condensation (Sano et al., 2020), CVD (Gutés et al., 2012; Tyurikova et al., 2020), sol-gel method (Gonçalves, 2018), soft lithography (Fu et al., 2018), hydrothermal methods (Zhen et al., 2019; Moreira et al., 2020), microwave methods (Henam et al., 2019), sonochemical (Gupta and Srivastava, 2019; Moreira et al., 2020), synthesis using plant extracts (Ogunyemi et al., 2019; Ranoszek-Soliwoda et al., 2019), and green synthesis (Gour and Jain, 2019; Irshad et al., 2020). On the other hand, macroscopic level materials can be trimmed down to NPs by different methods, including mechanical grinding (Sviridov et al., 2017; Haque et al., 2018), ball milling (Li Y. et al., 2020), lithography (Fu et al., 2018), vapor deposition (Choi et al., 2018), arc-plasma deposition (Ito et al., 2012; Takahashi et al., 2015), ion beam technique (Heo and Gwag, 2014; Yang J. et al., 2018), severe plastic deformation (Cui et al., 2018; Sarkari Khorrami et al., 2019), chemical etching (Wareing et al., 2017; Pinna et al., 2020), sputtering (Pišlová et al., 2020), and laser ablation (Pandey et al., 2014; Abid et al., 2020).

### Classification by Antibacterial Active Ingredients

Antibacterial nanomaterials can be categorized into a metal ion and oxide photocatalytic type according to antibacterial active ingredients. Metal ion antibacterial nanomaterials are metallic ions with antibacterial functions (Ag, Cu, Zn, Ni, Co, Al) loaded in a variety of natural or synthetic substrates. They can slowly release the antibacterial ion components to periphery tissues in order to achieve the antibacterial and bactericidal effects. Oxide photocatalytic antimicrobial materials commonly are TiO, ZnO, MgO, CdS, etc. and act in the catalysis of photocatalyst in which OH^–^ and H_2_O molecules oxidized to OH free radicals with strong oxidation capacity. Thus, these activated surfaces can inhibit and kill microorganisms that exists in the environment (Buzea and Pacheco, 2017).

## Preparation of Nanostructure on Titanium and Its Alloys

Titanium-based nanostructure (NS) materials have become the focus of current research because of their unique properties in optical, biological, and electrical fields (Miao et al., 2015; Gupta et al., 2018; Wei et al., 2020). Different preparation processes can be used to construct NS titanium surfaces, common nano-morphologies for titanium surfaces with antibacterial functions are nanotube and nano-coating forms. Titanium surfaces with different physical and chemical properties can influence the biological interactions and the adhesion of cells and bacteria (Zhou J. et al., 2018; Elbourne et al., 2019), which in turn affects the ability of early osseointegration and the risk of implant infection.

### Preparation of Nanotubes

Titanium dioxide nanotubes have received extensive attention because of their controllable size and highly ordered surface arrangement. Nanotubes have a larger specific surface area and storage space than other NS forms such as nanorods, nanospikes, and nanowires. These characteristics make it a good candidate among the other NS morphologies for the storage and release of antibacterial agents. The preparation of nanotube structures on the surface of titanium is mainly achieved by three methods: template synthesis (Jung et al., 2002; Lee et al., 2005), electrochemical anodization (Jun et al., 2012; Roman et al., 2014; Cao S. et al., 2018; Shang et al., 2019; Zhang et al., 2019d), and hydrothermal treatment (Tsai and Teng, 2004, 2006), they have different advantages and disadvantages listed in Table 1.

**TABLE 1:** Different nanotube formation processes.

Preparation technique	Shape and size	Characteristics	References
Template synthesis	(1) Tubular arrays or loose aggregates (2) Diameter:10∼500 nm (3) Length: Nanometer to micrometer	(1) Nanotubes with different diameters can be prepared (2) Removing the template may destroy the morphology of the nanotubes	Choi et al., 2017; Luo et al., 2018
Electrochemical anodization	(1) Highly ordered array of nanotubes (2) Diameter:10∼500 nm (3) Length:100 nm∼100 μm	(1) Highly ordered (2) Low degree of aggregation	Fathy Fahim et al., 2009; Zhao et al., 2014
Hydrothermal treatment	(1) Single or loose block tube (2) Diameter:2∼20 nm (3) Length: nanometer to micrometer	(1) Simple process (2) It can prepare small diameter nanotubes (3) Difficult to form nanotube arrays	Wang L. et al., 2014; Aal et al., 2015

#### Template Synthesis

The template synthesis method can be categorized into a hard template and a soft template method according to the nature of templating agent. The hard template method uses columnar single-crystal anodized aluminum oxide (AAO) (Hoyer, 1996; Ma, 2007; Newland et al., 2018) as a template to prepare nanotube structures by electrochemical deposition. The preparation process for the hard template is complicated, and the shape and size of the nanotubes are dependent on the size and shape of the template hole. Besides, the nanotubes destruction is possible during the separation step from the matrix template so unfortunately, it has poor reproducibility. In the soft template procedure surfactants used as templates (Bernal et al., 2012; Choi et al., 2017). Firstly, the surfactant should be mixed with water, titanium alkoxide, and other substances, then the polymerization takes place under certain conditions. After drying and calcination, the nanotube structure is prepared. The soft template method overcomes the shape and size dependence to the template holes, which is one of the big limitations of hard templates. However, due to the necessity of high temperatures in removing step of soft templates, the nanotubes may collapse.

Template synthesis can prepare nanomaterials upon design demands and obtain well-formed nanoarrays, but it still has some drawbacks. For example, the AAO template as a commonly used master plate is mechanically weak which makes it difficult to prepare metal templates with large areas (Masuda and Fukuda, 1995). Moreover, when using solvents to remove the organic compound templates, the structure of the nanomaterials may encounter some damages. Therefore, this synthesis method has the potential to be further optimized to synthesize utility materials with the desired functions using a simple operation (Bera et al., 2004).

#### Electrochemical Anodization

Anodizing is one of the most commonly used methods for preparing TiO_2_ nanotubes. The nanotube formation with a high aspect ratio and the excellent ordered condition can be attained by this method. The favorable nanotube size can be achieved by changing different factors (electrolyte composition, voltage, pH, anodizing time, etc.) (Gong et al., 2001; Ni et al., 2013; Wang L.N. et al., 2014; Khudhair et al., 2016; Song et al., 2017). Nanotube structures with tube dimensions in the range of 0.2-1000 μm length, 15–250 nm diameters and 10–70 nm thickness can be prepared with different anodic oxidation parameters. The main process of preparing titanium dioxide nanotubes by anodic oxidation are as follows: placing titanium foil or titanium sheet in a reaction cell (electrolytes are usually fluoride ions such as HF, NH4F, Macak et al., 2005; Alivov et al., 2009; Minagar et al., 2014) of two- or three-electrode system, and then a constant voltage application. The titanium sheet or foil is oxidized by the combined action of the electric field and fluoride ions. After some time, arrays of titanium dioxide nanotubes with uniform distribution, ordered arrangement and perpendicular to the substrate would be formed. The diameter of the nanotube can be controlled by the oxidation voltage (Bozkurt Çırak et al., 2017); the length of the nanotube is controlled by the combination of the oxidation time (Bhadra et al., 2020), the oxidation temperature (Mohan et al., 2020), and the pH value of the electrolyte (Cipriano et al., 2014); the smoothness of the nanotube, and the cleanliness of the surface are respectively dependent on the type of electrolyte and the water content (Sivaprakash and Narayanan, 2020).

#### Hydrothermal Treatment

Hydrothermal treatment is among the momentous methods for nanotube production (Harsha et al., 2011). This method has the potential to produce titanium dioxide nanotubes with suitable crystalline structures (Swami et al., 2010). In this procedure, usually TiO_2_ NPs were utilized as a titanium source to carry out a chemical reaction in a concentrated alkaline solution at high temperature. Subsequently, after ion exchange and calcination, the nanotube structure is reached (Wong et al., 2011). The hydrothermal method can synthesize nanotubes with different diameters and lengths by controlling the reaction conditions (Chi et al., 2007; Nakahira et al., 2010; Khoshnood et al., 2017; Mi et al., 2017). Hydrothermal synthesis is performed at high temperatures, and the rate of heating is a critical factor (Seo et al., 2001). Based on the rate of heating, the hydrothermal method can be divided into two categories: conventional and microwave hydrothermal procedures. In the traditional hydrothermal synthesis method, the sample is simply heated in a general water bath. However, its heating rate is slow and the reaction cycle is long. Additionally, the heating of the reaction system is not uniform. The reaction system is exposed to microwave radiation during the microwave hydrothermal synthesis method. Since microwave heating is a rapid method of heating, the temperature of the reaction system very rapidly increases which results in a significantly reduced reaction period and the more uniform heating (Ribbens et al., 2008; Liu et al., 2014; Meng et al., 2016).

Titanium dioxide nanotubes can be synthesized by template synthesis, electrochemical anodization, and hydrothermal treatment. Additionally, there are some studies on the fabrication of nanotube structures by plasma electrolytic oxidation. Among the various possible methods, electrochemical anodization is the most commonly used method. By controlling the variables of the electrochemical anodization method, it is feasible to fabricate nanotube structures that satisfy the research needs. It is of great importance to delicately control the release of antibacterial agents in nanotubes and regulate the optimal size of nanotubes in order to promote cell adhesion, proliferation, and differentiation.

### Preparation of Nano Coating

The formation of nano-scale coatings on the surface of titanium can endow new functionalities to the surface. There are various methods for nano-coatings preparation. the conventional surface coating technologies are chemical vapor deposition (CVD) and physical vapor deposition (PVD). Newly developed methods include sol-gel, spin coating, plasma spraying, layer-by-layer self-assembly, and electrophoretic deposition, these methods are listed in Table 2.

**TABLE 2:** Various nano-coating preparation methods and the related characteristics.

Preparation technique	Characteristics	References
Chemical vapor deposition	(1) Lower equipment cost	Son et al., 2016
	(2) Controllable coating density and purity	
	(3) Coatings deposition on complex shapes	
	(4) The coating is uniform and dense	
	(5) Firmly combined with the base materials	
Physical vapor deposition	(1) Simple production process	Hübsch et al., 2015
	(2) No pollution, less consumables	
	(3) The coating is uniform and dense	
	(4) Firmly combined with the base materials	
Spin-on deposition	(1) Simple production process	Nguyen et al., 2020
	(2) Low preparation cost and low pollution	
	(3) Accurate and controllable coating thickness	
Sol-gel method	(1) Simple production process and low equipment requirements	Hübsch et al., 2015
	(2) Can be prepared at room temperature	
	(3) Large area coating	
	(4) High purity and homogeneous coating	
Plasma spraying	(1) Simple production process	Mahade et al., 2019
	(2) Suitable for multiple materials	
	(3) Coating with low porosity, high density and smooth feature	
Layer-by-layer self-assembly	(1) Simple production process without any need to special equipment	Elmi et al., 2019
	(2) Suitable for multiple materials, including polymer materials	
	(3) Can precisely control the coating structure and size	
Electrophoretic deposition	(1) Simple production process and convenient operation	Pishbin et al., 2013
	(2) Low preparation cost	
	(3) Accurate thickness control, chemical composition, and porosity	
	(4) Low temperature requirements	

#### Chemical Vapor Deposition

In the CVD method a single substance or compound containing one or more gas phases of elements is utilized to perform a chemical reaction on the substrate surface and produce a coating. In recent decades, even inorganic coatings can be produced through CVD technologies. Moreover, these methods can be used to purify various substances and precipitates, single-crystal, polycrystalline, and other inorganic thin-film materials (Jin et al., 2013; Manawi et al., 2018). Based on the influential parameters and chemical interactions, CVD methods can be divided into ambient pressure (Jang et al., 2015), low-pressure (Gao et al., 2011; Umrao et al., 2017), thermal (Zeng et al., 2018), ultra-high vacuum (Multone et al., 2008), and organic metal CVD methods (Maury and Senocq, 2003; Gong et al., 2013). Various oxide coatings, nitrides, and metal nano-coatings can be prepared by CVD methods based on the material and the required properties through its varied techniques (Delfini et al., 2017).

#### Physical Vapor Deposition

In the PVD technology, a physical phenomenon is used to vaporize the surface of source material (solid or liquid) into gaseous atoms, molecules, or into ions by ionization under vacuum condition. After the vaporization step, a low-pressure gas (or plasma) is implemented and a functional thin film is deposited on the substrate surface (Chouirfa et al., 2019). The PVD technology can deposit not only metallic and alloy films but also it is capable of depositing ceramics and polymers. The main PVD methods are vacuum evaporation, sputtering deposition, and ion plating.

Vacuum evaporation uses laser and electron beam heating to evaporate the material source into atoms or ions, and subsequently deposits atoms or ions onto the surface of the substrate to form a coating (Kumar et al., 2009; Garbacz et al., 2010). The resultant coating from this method has relatively large pores and poor adhesion to the substrate (Xingfang et al., 1988; Scott et al., 2003; Krysina et al., 2020). Sputter plating uses the base material as the anode and the target material as the cathode. By using the sputtering deposition effect, the argon ions generated by argon ionization knocks out the target material atoms and deposits them on the surface of the base material. The characteristic of this kind of coating is the presence of a few pores, but it can combine with the substrate more efficiently (Ratova et al., 2017; Makówka et al., 2019; Zhang et al., 2019a). Ion plating is involved with the ionization of gasses or vaporized substances under vacuum conditions, during the bombardment of gas ions or vaporized material ions, evaporates, or other reaction products are deposited on the substrate. The coating prepared by this method is uniform and dense, basically free of pores with a strong binding with the substrate (Li et al., 2017c; Zhang et al., 2018a; Tian et al., 2019).

With the development of PVD methods, many advanced PVD-based technologies have been derived which facilitate the production of high-quality nano-coatings. These new developed technologies include activated reactive evaporation (Bulla et al., 2004; Biju et al., 2009; Yuvaraj et al., 2010), activated reactive sputtering (Alajlani et al., 2016, 2017), activated reactive ion plating (Xin et al., 2000), magnetron sputtering (Lelis et al., 2019; Avino et al., 2020; Vuchkov et al., 2020), magnetron sputtering pulsed laser deposition (MSPLD) (Endrino et al., 2002; Jones and Voevodin, 2004), ionized magnetron sputtering (Kusano et al., 1999; Tranchant et al., 2006), pulsed laser deposition (PLD) (Paneerselvam et al., 2020; Wang et al., 2020), and etc.

#### Sol-Gel Method

Sol-gel technology uses some compounds with high chemical activity as precursors. After the raw materials are uniformly mixed in the liquid phase, hydrolysis and condensation chemical reactions are carried out to form a stable sol. It reacts with water in a certain solvent and forms a sol through hydrolysis and polycondensation interactions. After the sol is aged, the colloidal particles slowly polymerize to form a gel with a three-dimensional grid structure. After the gel is dried, sintered, and solidified on the surface of the substrate, the NS coating would be achieved (Antonelli and Ying, 1995; Kim et al., 2004).

#### Spin-on Deposition

**FIGURE 3:**
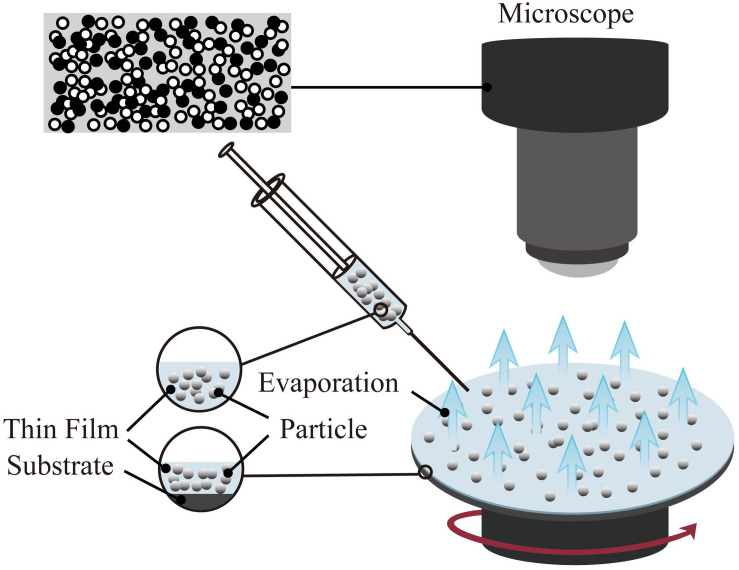
Schematic diagram of spin coating method(the target particles are applied onto the substrate, and then it accelerated to a high angular velocity to simultaneously spread the liquid over the entire surface and evaporate the solvent to achieve the target thickness).

One of the coating preparation methods entitled as the spin coating is able to precisely control the thickness. However, the size of the substrate is limited by the size of the spinning device (Abu-Thabit and Makhlouf, 2020). The thickness of the coating prepared by the spin coating method is in the range of 30 and 2000 nm. The typical spin coating method is mainly divided into three steps: glue dispensing, high-speed rotation, and drying. First, the spin-coating droplets are injected onto the surface of the substrate. Then, the spin coating solution is spread on the surface of the substrate through high-speed rotation to form a uniform film. Finally, the remaining solvent is removed by drying; hence the stable coating is obtained. In the process of preparing the coating by spin coating, high-speed rotation, and drying are the key steps to control the thickness, structure, and performance of the coating. The schematic of the spin coating method is shown in Figure 3.

Spin coating technology has been successfully applied in the fields of optics (Chtouki et al., 2017; Al-Douri et al., 2018) and electricity (Oytun and Basarir, 2019; Yildiz et al., 2019). Simultaneously, the spin coating method is also used in the preparation of functional thin films in the fields of biology and medicine. For example, a hydrophilic or hydrophobic film is produced on the surface of the base material to achieve the purpose of antibiosis (Kaviyarasu et al., 2017; Huang Y. et al., 2019; Li H. et al., 2020) and anti-corrosion (Kim et al., 2013; Akram et al., 2020).

#### Plasma Spraying

**FIGURE 4:**
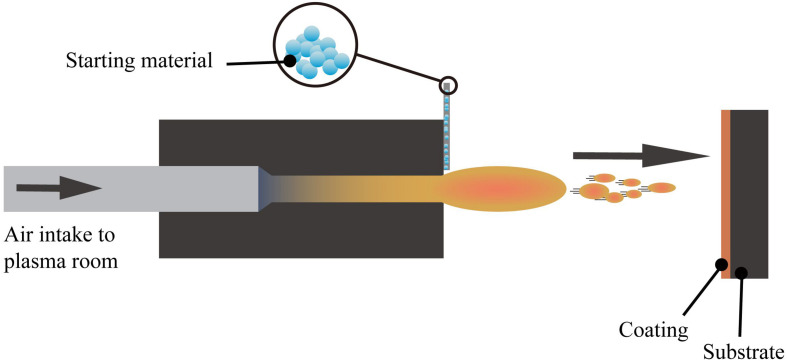
The layout of the plasma spraying setup (Viana et al., 2017).

Plasma spraying technology uses a plasma arc that driven by direct current as a heat source to heat ceramics, alloys, metals, and other materials to a molten or semi-molten state (Fauchais and Montavon, 2007; Mostaghimi and Chandra, 2007). These materials are sprayed onto the surface of the base material at high speed, and a firmly attached surface coating is formed, this process is schematically presented in Figure 4. Plasma spraying is a fundamental nano-coating preparation process. This process has high stability and excellent controllability, and a variety of materials can be used to prepare the coating. At the same time, the prepared coating has low porosity and high deposition efficiency and it is suitable for preparing high melting point metal and ceramic coatings (Cheang and Khor, 1996; Huang et al., 2014; Goudarzi et al., 2018).

The plasma sprayed functional coatings can improve the thermal insulation, anti-oxidation, and surface optical performance of the base material. With further research on functional coatings prepared by plasma spraying, some advances have made in the biomedical field. For example, the preparation of silver-containing coatings with antibacterial effect on the surface of CoCr alloys using vacuum plasma spraying technique (Liang et al., 2020). Also, the HAp coating on the Ti6Al4V surface using the axial suspension plasma spraying method (Hameed et al., 2019), and the HAp coating prepared by the micro-plasma spray method (Wang et al., 2017), both presents enhanced biological performance than the traditional plasma spraying methods.

#### Layer-by-Layer Self-Assembly

**FIGURE 5:**
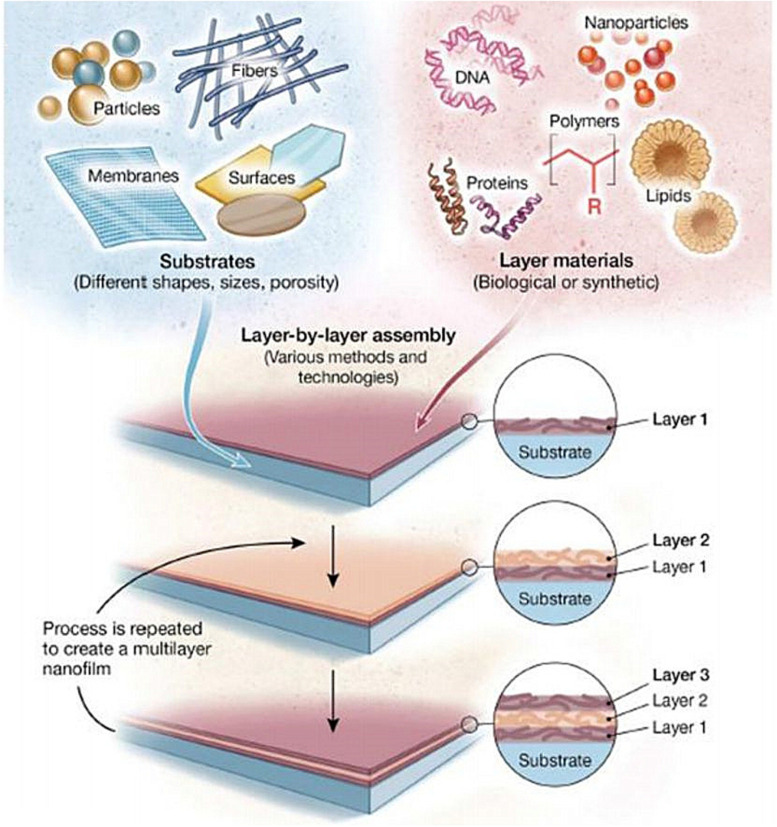
Schematic illustration of LbL assembly technology, which can load different types of materials on different types of substrates (Zhang et al., 2018d).

Layer-by-layer self-assembly technology is a relatively new technology in recent years and is widely used in the biology, materials, and nanoscience fields. As shown in Figure 5 (Zhang et al., 2018d), this technology can assemble a variety of materials (polyelectrolytes, small organic molecules, NPs, etc.) and can precisely control the surface structure and size of the coating. Nanomaterials with ordered structure prepared by self-assembly technology show unique properties. Self-assembly technology is currently a hotspot in the field of nanomaterial research.

Layer-by-layer self-assembly technology can be used to fabricate filtration membranes (Rajesh et al., 2016), sensors (Fernandes et al., 2011), and optoelectronic devices (Eom et al., 2017). Besides, it is able to produce antibacterial coatings (Wu et al., 2015; Huang J. et al., 2019; Li D. et al., 2019; Xia et al., 2019) or drug controlled release coatings (Cao M. et al., 2018; Silva et al., 2018; Zhou W. et al., 2018; Sun et al., 2020; Wu et al., 2020). Hence, this technology seems to would have broad application prospects in the biomedicine field.

#### Electrophoretic Deposition

Electrophoretic deposition is the directional movement of charged particles in the direction of the electrode under the action of an electric field. The outer layer of ions exerts pressure on the charged particles, forcing the particles to gather near the electrode and lead them to deposit (Besra and Liu, 2007). The method can prepare a coating with a thickness of 0.1–100 μm, which can meet the coating thickness requirements of various medical implant materials. Electrophoretic deposition techniques with many advantages can be used to prepare bioceramic coatings on metallic substrates (Boccaccini et al., 2010; Avcu et al., 2019; Alaei et al., 2020).

Mahlooji et al. (2019) prepared the chitosan-bioactive glass (CS-BG) nanocomposite coating on the surface of Ti-6Al-4V alloy by the electrophoretic deposition. The coating has excellent adhesive strength with the base material which can effectively promote the formation of apatite, and also it has a favorable biological activity. In addition, there are several related studies on the application of electrophoretic deposition technology to prepare bioactive glass coatings with various biological activities (Ur Rehman et al., 2018; Ghalayani Esfahani et al., 2019). There are also studies about the fabrication of antibacterial coatings by this method (Braem et al., 2017; Bakhshandeh and Amin Yavari, 2018; Ning et al., 2019; Thinakaran et al., 2020). A study used copper and chitosan to synthesize copper(II)-chitosan[Cu(II)-CS] complex coating, which has an excellent antibacterial effect on both Gram-positive and Gram-negative bacteria. In addition, Human osteoblast-like cells were cultured on the coating surface, which confirmed that the Cu (II) -CS coating had no cytotoxic effect (Akhtar et al., 2020). Furthermore, the results of a study about the chitosan hydrogel membrane (CHM) production by electrophoretic deposition show that the resultant coating can effectively promote the adhesion and growth of L-929 mouse fibroblast cells, and has good biocompatibility. It can also be loaded with suitable cells as a graft and is valuable from the application point of view (Li et al., 2017d).

Nanocoatings can be manufactured by various methods such as vapor deposition, different kinds of spraying, electrodeposition, etc. By considering the existing coating technologies, different manufacturing methods can be selected according to different needs. Adding the appropriate nanomaterials and precise adjustment of the coating parameters leads to manufacturing the desired nano-coatings. Compared with conventional coatings, nano-coatings have excellent mechanical properties, such as lower porosity, higher bond strength, higher hardness, oxidation resistance, corrosion resistance, etc. (Taylor and Sieradzki, 2003; Ranjbar and Rastegar, 2011; Lin et al., 2013). Therefore, the application of surface coatings in different fields will be very beneficial. In addition, the nanosurface coatings with antibacterial properties will be the great help in solving the implant-related infections and antibiotic resistance issues in clinical practice (Kose and Ayse Kose, 2015; Yilmaz and Yorgancioglu, 2018). There are still many problems related to nanocoatings that need further research and discussion, such as the dispersion technique and stability of nanoparticles in the coating medium, the different properties of various types of nanoparticles, and their possible applications.

## Nanomaterials Loading

Nanomaterials can be used as antibacterial agents on the surface of titanium or titanium alloys and they have a potential to effectively improve the bactericidal properties (Chen et al., 2014; Xin et al., 2019; Xu J.W. et al., 2019). Most of the studies were focused on the metallic nano-antibacterial agents on the surface of titanium and its alloys (Liu et al., 2015b; Gunputh et al., 2018; Cheng et al., 2019). In this regard, the common metal particles are silver, zinc, copper, etc. (Vimbela et al., 2017). The NPs are tiny particles with a particle size in the range of 1–100 nm. The specific properties of NPs like large specific surface area, small size effect, and quantum size effect makes them an ideal option. Therefore, nanomaterials have a series of excellent properties, which improves the bactericidal effects of antibacterial agents in comparison to traditional antibacterial agents (Mi et al., 2018; Guo et al., 2020).

### Metallic Antibacterial Agents

Metallic antibacterial agents have exceptional research value because of their strong antibacterial ability, good biocompatibility, and excellent stability (Ahmed et al., 2016). Inorganic antibacterial agents are usually present on the surface of titanium substrates in the form of NPs. They can be fixed on the surface of the titanium substrate by using a carrier or coated on the titanium substrate’s surface to prepare a nano-coating (Kheiri et al., 2019).

#### Ag

Silver has many advantages in a broad-spectrum of antibacterial activity (Yang Z. et al., 2018; Wang L. et al., 2019). These advantages make it the most studied and widely used metal-based antibacterial agent. Compared with traditional silver, silver NPs have a larger specific surface area, which significantly enhances their antibacterial ability. In the existing reports, the antibacterial mechanism of silver NPs mainly includes: destroying the structure of bacterial membranes, releasing silver ions and generating ROS to destroy enzymes in the oxidation respiratory chain, and regulating the signal transduction pathway of bacteria (Park et al., 2009; Tang and Zheng, 2018). Unfortunately, silver NPs can cause significant cytotoxicity above a specific dose range. The silver ions released by the silver NPs are highly mobile, and their entrance into living cells with high concentrations can kill healthy cells (AshaRani et al., 2009). The silver element forms which served as antibacterial are Ag2O NPs (Chen et al., 2017; Sarraf et al., 2018a; Lv et al., 2019) and Ag NPs (Cheng et al., 2019; Pruchova et al., 2019; Surmeneva et al., 2019). The silver NPs that were loaded on the surface of the titanium substrate to produce NS, showing different antibacterial effects and performance that is listed in Table 3.

**TABLE 3:** Antibacterial effects and other related information about different silver-containing nanomaterials.

Materials	Base materials	Manufacturing technique	Nanostructure and Size	Antibacterial effect/Antibacterial rate	Cytotoxicity	Cells	References
Ag-CACS-Ti	Cp-Ti	*In situ* reduction	Nanoparticles	*E. col*i: 99.8% *S. aureus*: 99.9%	No	L929 cells	Cheng et al., 2019
Ag-TNTs	Ti-6Al-4V	Anodization+Chemical reduction	Nanoparticles: 102 ± 21 nm	*S. aureus*: 99.15%	–	–	Gunputh et al., 2018
Ag-HA	Ti-6Al-4V	Laser process	Nanoparticles: 20∼30 nm	*S. aureus*: 77.59%	–	–	Liu and Man, 2017
Ag-HA-CS	Cp-Ti	Pulse electrochemical deposition	Nanoparticles: 303–321 nm	*E. coli*: 100% *S. aureus*: 100% *C. albicans*: 100% *P. aeruginosa*: 100%	No	BMSCs	Wang X. et al., 2019
Ag-PDA-TNTs	Cp-Ti	electrochemical anodization + *in situ* reduction	Nanoparticles: 13.5 ± 4.8 nm	*E. coli*: 54 ± 3.7%/14 days	–	–	Xu et al., 2017
Ag-PDA-TNTs	Ti-7.5Mo	Anodization + polydopamine assisted immobilization technique	Nanoparticles	*C. albicans*: 100%/48 h *S. aureus*: 100%/48 h	No	ADSCs	Rosifini Alves Claro et al., 2018
Ag-GO	Ti-67IMP	Physical vapor deposition magnetron sputtering + electrochemical anodization + spin coating	Nanoparticles	*E. coli*: 97.56%/24 h *S. aureus*: 98.15%/24 h	No	hFOB cells	Rafieerad et al., 2019
Ag-Ti	Cp-Ti	Target-ion induced plasma sputtering + Ag sputtering	Nanoparticles: 25 ± 5 nm	*E. coli*: 100%/12 h *S. aureus*: 100%/12 h	Yes	L929 fibroblast cells	Kim et al., 2018
TAN/TAP	Ti-Si	Vacuum arc remelting + sol-gel method	Nanoparticles: ∼2 μm	*E. coli*: 98-100%/24 h *S. aureus*: 100%/24 h	No	L929 fibroblast cells; U-2OS human osteosarcoma cells	Horkavcová et al., 2017
Ag-Sr-HA coating	Cp-Ti	Hydrothermal method	Nanoparticles: ∼100 nm	*E. coli*: 99%/24 h *S. aureus*: 95%/24 h	No	MG63 cells	Geng et al., 2016
Ag-HA coating	Cp-Ti	Electrostatic spraying	Nanorods: length: 50 nm, diameter: 20 nm	*E. coli*: 100%/24 h	No	Osteoblast	Gokcekaya et al., 2017
Ag film	Ti-6Al-4V	Thermal annealing + DC sputtering	Thickness: 20 nm	*E. coli*: 100%/24 h *S. aureus*: 100%/24 h	Yes	NIH3T3 fibroblast cells	Patil et al., 2019
Ag-TiN multilayers	Titanium alloy	Multi-arc ion plating	Thickness: 120 nm	*E. coli*: 99.88%/24h	Yes	MC3T3-E1 cells	Zhao et al., 2019
NiTiAg coating	Cp-Ti	Electrodeposition + anodization	–	*S. aureus*: 64.52% *S. epidermidis*: 92.35%	–	–	Huang et al., 2017
Ag-DLC coating	Cp-Ti	Thermionic vacuum arc	Nanoparticles: 20 ± 3 nm	*S. aureus*: 94.2%/24 h	–	–	Mazare et al., 2018
Ag-CDHA	Ti-6Al-4V	Microwave assisted technique	Nanoparticles: 25.1–40.4 nm	*E. coli*: 83.68%/24 h *S. aureus*: 82.31%/24 h	No	MC3T3-E1 cells	Sikder et al., 2018
Ag-Pdop hybrid films	Ni-Ti	One-step immersion method	Nanoparticles: average 11.75 nm	*S. aureus*: >99%/24 h	No	hBMSCs	Yin et al., 2019
CS-Ag-MoS2-Ti	Cp-Ti	Ultraviolet light induced reduction	–	*E. coli*: 99.77%/24 h *S. aureus*: 98.66%/24 h	No	NIH3T3-E1 cells	Zhu et al., 2020
Ag2O-Ta2O5 NTs	Ti-6Al-4V	Anodization + physical vapor deposition	–	*E. coli*: 100%/2 h	No	Osteoblast	Sarraf et al., 2018b
Ag2O-TNTs	Ti-6Al-4V	Anodization + physical vapor deposition	–	*E. coli*: 100%/2 h	No	Osteoblast	Sarraf et al., 2018a
Ag2O-TiO2 coating	Cp-Ti	Micro-arc oxidation	–	*S. aureus*: 99%/24 h	No	NIH3T3-E1 cells	Lv et al., 2019
Ag2O-TNTs	Cp-Ti	Hydrothermal treatment	–	*S. aureus*: 100%/10 days; 84%/60 days	No	NIH3T3-E1 cells	Chen et al., 2017

*TAN, Titania sol + AgNO_3_; TAP, Titania sol + Ag_3_PO_4_; DLC, diamond-like carbon; CDHA, calcium deficient hydroxyapatite.*

A large number of experiments have proved that silver NPs can effectively exert antibacterial properties. Silver NPs have a good killing effect on Gram-positive cocci represented by *Staphylococcus aureus* and Gram-negative bacilli represented by *Escherichia coli* (Deshmukh et al., 2018; Subramaniyan et al., 2019). Currently, the biggest challenge is to enable the stable release of silver at a suitable concentration on the surface of metal implants (Yang Z. et al., 2019; Zhu Y. et al., 2019; Lai et al., 2020). The nanotube structure, which is prepared on the surface of the titanium substrate and loaded with silver or Ag_2_O NPs, is one of the solutions. Then, based on this structure, a controlled release coating (such as a polydopamine coating) is prepared, the controlled release phenomenon helps to achieve a long-term antibacterial effect (Gao et al., 2019). Compared with silver NPs, the amount of Ag^+^ released from Ag_2_O NPs is lower, which may be due to the role of the oxide barrier. According to reports, Ag_2_O NPs have a larger total surface-to-volume ratio, which increases their contact area with microorganisms (Morones-Ramirez et al., 2005). Compared with Ag NPs, Ag_2_O NPs can also permeate or attach Ag^+^ ions to the bacterial membrane to achieve better killing of bacteria, while reducing the negative impact on mammalian cells (Sarraf et al., 2018b). In addition, the antibacterial effect of silver NPs *in vivo* is noteworthy. Guan et al. (2019). develop a novel surface strategy involving the formation of polydopamine (PDA) and silver (Ag) nanoparticle-loaded TiO_2_ nanorods (NRDs) coatings on Ti alloy. *In vitro* antibacterial experiments showed that Ag-TiO_2_@PDA NRDs coatings have antibacterial effects on Methicillin-resistant Staphylococcus aureus on days 7 and 14 according to the bacterial counting method. The efficiencies were 88.6 ± 1.5% and 80.1 ± 1.1%, respectively. The material was then implanted into the tibia of a rat model of osteomyelitis. After four weeks, the results of X-ray, micro-CT, and H&E staining showed that MRSA in the tibia of rats could be killed by Ag+, confirming that the material also had good antibacterial activity *in vivo*.

Moreover, with the release of silver NPs, the antibacterial properties of the implant material surface will gradually weaken hence it would be unable to have a long-term and stable antibacterial effect. Therefore, controlling the silver NPs release process and reducing the cytotoxic reactions caused by high concentrations of silver ions is one of the main research directions.

#### Cu

Copper is an essential trace element for the human body. Copper plays an important role in maintaining the normal hematopoietic function of the human body, promoting the formation of connective tissue and maintaining the health of the central nervous system. At the same time, copper has strong antibacterial properties and is not prone to drug resistance. Copper NPs can play an essential role in inhibiting infection and forming bone matrix by releasing copper ions (Liu et al., 2019; Mou et al., 2019; Anitha and Muthukumaran, 2020; Lv et al., 2020). As a redox metal (Tripathi and Gaur, 2004; Rauf et al., 2019), it can catalyze the formation of ROS, and at the same time, can destroy the permeability of bacterial membranes, resulting in the leakage of reducing sugars and proteins from cells. These mechanisms caused fatal damage to the bacteria. The antibacterial properties and characteristics of Cu NPs and NS containing Cu NPs are shown in Table 4.

**TABLE 4:** Antibacterial effects and other information of different copper-containing nanomaterials.

Materials	Base materials	Manufacturing technique	Nanostructure	Antibacterial effect/Antibacterial rate	Cytotoxicity	Cells	References
Cu NPs coating	Cp-Ti	Laser ablation	NPs: ∼10nm	*S. aureus*: 83.21%/24 h		–	Fernández-Arias et al., 2020
Cu NPs coating	Cp-Ti	Plasma immersion ion implantation and deposition	Nanoparticles	*E. coli*: 50 ± 7%/24 h *S. aureus*: 90 ± 1%/24 h	No	rBMMSC	Yu et al., 2016
CuO-TiO2 coating	Cp-Ti	Magnetron sputtering	NPs: ∼100 nm	*S. aureus*: >99%/24 h	No	Osteoblasts	He et al., 2017
3Cu-HA coating	Ti-6Al-4V	Electrophoretic deposition	Nanoparticles: 50∼100 nm	*E. coli*: MIC: 700 μg*ml^–1^/20 h *S. aureus*: MIC: 1400 μg*ml^–1^/20 h	No	MG63 cells	Hadidi et al., 2017
5Cu-HA coating	Ti-6Al-4V	Electrophoretic deposition	Nanoparticles: 50∼100 nm	*E. coli*: MIC:300 μg*ml^–1^/20 h *S. aureus*: MIC:700 μg*ml^–1^/20 h	Yes	MG63 cells	Hadidi et al., 2017
2.5Cu-HA coating	Ti-6Al-4V	Laser deposition	Small aggregates: 200∼1000 nm	*E. coli*: reduce growth to 42% /24 h *S. aureus*: reduce growth to 75% /24 h	No	NIH3T3-E1	Hidalgo-Robatto et al., 2018
Cu-Ti-O NTAs	Cp-Ti	Anodizing magnetron-sputtered	–	*S. aureus*: >90%/28 d	No	EA. hy926 cells	Zong et al., 2017

#### Zn

Zinc is one of the important trace elements in the human body and plays a vital role in the growth and development of bones (Zhu et al., 2018). It was known that Zn can enhance the expression of M2 marker genes and proteins in macrophages. The adhesion, proliferation, and expression of osteoblast-related genes are increased by Zn (Zhang et al., 2018c; Chen et al., 2020). Zinc NPs are non-toxic and have a higher affinity to bacteria than ordinary zinc, which can lead to better antibacterial effect. In the current research on the antibacterial mechanism of zinc NPs, it has been found that zinc ions can destroy bacterial membranes and promote the production of ROS, thereby achieving antibacterial effects. Related research and results are shown in Table 5. To confirm the antibacterial effects and the feasibility of clinical applications Zinc-containing nanocoatings were studied, the results have shown excellent antibacterial effects both in vitro and in vivo antimicrobial experiments. Li et al. (2017b) prepared hybrid ZnO/poly-dopamine/arginine-glycine-aspartate-cysteine nanorod arrays on the titanium surface using atomic layer deposition and hydrothermal methods. The material was implanted into the femur of the rabbit model infected with *S. aureus*. After four weeks, femurs from animal models were tested by H&E staining and Giemsa staining, the zinc-containing nanorod arrays were less infected than the control soft tissues and bones, demonstrating that the release of zinc ions can play an effective anti-infective role.

**TABLE 5:** Different antibacterial properties and biocompatibility of zinc-containing coatings.

Materials	Base materials	Manufacturing technique	Nanostructure	Antibacterial effect/Antibacterial rate	Cytotoxicity	Cells	References
ZnO-Sr-OPDA-TNTs	Cp-Ti	Anodization hydrothermal treatment + atomic layer deposition	ZnO film:thickness<2 nm	*E. coli*: 87%/12 h *S. aureus*: 91%/24 h	No	MC3T3-E1 cells	Zhang K. et al., 2017
ZnO-CS-CNTs-Ti	Cp-Ti	Electrophoretic deposition atomic layer deposition	ZnO film thickness: <10 nm	*E. coli*: 73%/24 h *S. aureus*: 98%/24 h	Yes	MC3T3-E1 cells	Zhu et al., 2017
ZnO-TiO2 coating	Cp-Ti	Hydrothermal low temperature liquid phase	Nanoparticle diameters: 50 ± 8∼80 ± 9 nm	*E. coli*: 93.2%/12 h *S. aureus*: 97.5%/24 h	No	MC3T3-E1 cells	Pang et al., 2019
Zn-Ti nanowires	Cp-Ti	Sol-gel + alkali heat	–	*S. aureus*: 66.58%/3 days *P. gingivalis*: 45.02%/3 days *A. actinomycetemcomitans*: 53.42%/3 days	No	MC3T3-E1 cells	Shao et al., 2020
Zn-CHI-GEL multilayer films	Cp-Ti	LBL self-assembly	Film thickness: 14.91 ± 0.97∼ 17.72 ± 0.63 nm	*E. coli*: 47.37%/24 h *S. aureus*: 52.94%/24 h	Yes	Osteoblasts	Karbowniczek et al., 2017
ZnO-TiO2 coating	Cp-Ti	Micro-arc oxidation	Nanoparticles: Average 30 nm	*S. aureus*: 51.4 ± 14.7%/24 h	No	MC3T3-E1 cells	Zhang et al., 2018b
ZnO-Ti coating	Cp-Ti	Micro-arc oxidation	–	*E. coli*: 48.08%/24h		–	Zhang et al., 2019c
ZnO-PPy-HA coating	Cp-Ti	Electrochemical deposition	Nanoparticle: Average 243 nm	*E. coli*: 63.5%/12h *S. aureus*: 72.8%/12h	No	BMSCs	Maimaiti et al., 2020
Zn-HA coating	Ti-6Al-4V	Co-precipitation + flame spraying	50–200 nm	*E. coli*: 99.9%/3 h	No	WST-1 cells	Yang et al., 2017
Zn-HA coating	Ti-6Al-4V	Plasma spraying	–	*E. coli*: 63.5%/7 days *S. aureus*: 36.6%/7 days	No	Saos-2 cells	Sergi et al., 2018

#### Au

Gold nanoparticles (Au NPs) as an antibacterial agent have been demonstrated in many researches (Grandi et al., 2011; Samanta et al., 2019). It has also some other surface functions such as photocatalysis, photothermal effect, and ROS-stimulating activity (Xia et al., 2015). Au NPs can also achieve antibacterial effects by destroying the cytoplasmic membrane of bacteria (Li et al., 2016). Au NPs are non-toxic and highly light stable, which can also be used as a probe to accurately locate biological macromolecules on the cell surface and within the cell, and can also be used for immunohistochemical localization.

Yang T. et al. (2019) prepared a gold nanorods (GNR) structure on the Ti surface by layer-by-layer self-assembly method. Also, they evaluate the photothermal antibacterial efficiency of Ti-GNR under near-infrared radiation (NIR) with a wavelength of 808 nm. The alamarBlue^TM^ assay was used to detect the number of viable bacteria on different samples. The results showed that the antibacterial rates of Ti-GNR on *S. aureus*, *S. epidermidis*, *E. coli*, and *P. aeruginosa* were 45.72, 56.94, 14.61, and 20.24%, respectively. The NIR-treated Ti-GNR-NIR surface had antibacterial rates of 26.31, 31.84, 61.82, and 66.74%, respectively. The results show that the Ti-GNR surface after near-infrared radiation has high antibacterial activity against *E. coli* and *P. aeruginosa*. At the same time, the cell culture results showed that the Ti-GNR and Ti-GNR-NIR surfaces had lower cytotoxicity to MC3T3-E1 cells. Xu W. et al. (2019) prepared TiO_2_ nanotube arrays (TNT) on Ti plates by anodizing, and then loaded gold NPs (Au NPs) into TNT. Under visible light, the antibacterial ability of nanotubes loaded with gold NPs against anaerobic bacteria was evaluated. The experimental results show that the average antibacterial efficiency of TNT materials loaded with Au NPs is above 85%, and the highest antibacterial rates for *F. nucleatum* and *P. gingivalis* can reach 92.13 and 97.34%.

#### Ni

Nickel is an indispensable element in the human body, and its content is in minimal range in the human body. Nickel maintains the structural stability and metabolism of biological macromolecules. Lack of nickel can cause diabetes, uremia, kidney failure, and other diseases. Studies have shown that Ni^2+^ can effectively kill bacteria (Yasuyuki et al., 2010), but excessive Ni^2+^ will cause cytotoxicity (Lü et al., 2009; Hang et al., 2012). A recent study showed that Ni^2+^ released from NiTi alloys could exhibit antibacterial properties (Ohtsu et al., 2017). However, due to slight Ni^2+^ release from the NiTi alloy, its antibacterial ability is relatively weak. Therefore, in nanometric range the specific surface area of the NiTi alloy is increased, and the release amount of Ni^2+^ is increased, thereby enhancing its antibacterial ability. Hang et al. (2018) produced Ni-Ti-O NPs with different lengths (0.55–114 μm) on NiTi alloys by anodization. The antibacterial effect of different samples to *S. aureus* was determined by the plate counting method. It was found that the antibacterial rate increased as the anodizing time increased. When the length of Ni-Ti-O NPs exceeds 11 μm, the antibacterial rate can reach 100% along with its excellent biocompatibility.

Liu et al. (2018) also used anodizing method to prepare Ni-Ti-O NPs on NiTi alloy and studied the effects of different annealing temperatures (200, 400, 600°C) on antibacterial properties and biocompatibility. The length of the NPs is 2.05–2.78 μm. The results show that the antibacterial rate of the smooth NiTi alloy surface is only 36%, and the antibacterial rate of the surface after anodizing reaches 84%. After annealing at 200°C, the antibacterial rate of Ni-Ti-O NPs was close to 100%. Ni-Ti-O NPs annealed at 200–400°C all showed good cell compatibility. Therefore, the preparation of NPs with different sizes on the surface of NiTi alloy can effectively enhance the antibacterial effect of the material by increasing the specific surface area.

An enormous number of studies on different inorganic nanoparticles (Ag, Cu, Zn, Au, Ni) indicate that Ag NPs as antibacterial agents have very efficient antibacterial performance compared to other antibacterial agents. At present, the mechanism of antibacterial actions of silver and silver ions is still under controversy. As mentioned previously, Ag NPs stimulate the generation of ROS and induce high oxidative stress, which is thought to be the foremost antibacterial mechanism (Park et al., 2009; Siritongsuk et al., 2016). Under normal circumstances, ROS generated in the cell receives a restriction that can be eliminated by antioxidants (Ramalingam et al., 2016). The antibacterial effect of Ag NPs stems from the dehydrogenase inactivation in the oxidative respiration chain along with excessive ROS generation. These circumstances inhibit oxidative respiration and the natural growth of the cells (Su et al., 2009; Quinteros et al., 2016). In addition, two antibacterial mechanisms, contact killing, and ion-mediated killing are widely accepted. Ag NPs can anchor to the bacterial cell wall and infiltrate it, which can cause physical changes to the bacterial membrane (bacterial membrane damage, leakage of bacterial contents) and ultimately lead to bacterial death (Khalandi et al., 2017; Seong and Lee, 2017). The primary antibacterial form of Ag NPs is the silver ion (Ag^+^) (Liu and Hurt, 2010; Le Ouay and Stellacci, 2015), and the target of Ag ^+^ has been identified as a number of molecules [DNA, peptides (membrane-bound or inside the cell) or cofactors] (Le Ouay and Stellacci, 2015). The interaction of Ag NPs with cellular structures or biological molecules will result in impaired bacterial function and ultimately death. Antibiotics are usually used to attack particular molecules of certain bacteria, but silver ions react with all the nearby molecules, thus having a wide-spectrum antibacterial effect. Silver does not react with water, but can easily dissolve in water with the presence of an oxidizing agent (oxygen), which is termed oxidative dissolution. The oxidative dissolution of silver is also an important mechanism for its antibacterial action (Molleman and Hiemstra, 2015), through the above mentioned multiple mechanisms, which directly or indirectly lead to the ability of Ag NPs to exert efficient antibacterial effects.

The issue of cytotoxicity of Ag NPs still needs further studies since it depends on lots of factors, including the shape of NPs, dimension, concentration, etc. (Attarilar et al., 2020), since high concentration of Ag NPs may be cytotoxic. Moreover, some other studies demonstrated the influence of NPs’ size on cytotoxicity. Several studies have shown that Ag NPs with the small size range (<20 nm) may cause varying degrees of cytotoxic effects, while large or condensed particles (>100 nm) sometimes do not have any considerable adverse outcomes (Marambio-Jones and Hoek, 2010; Yang et al., 2012; Rizzello and Pompa, 2014; Foldbjerg et al., 2015). Traditionally, antibiotics targeting specific infections or classes of bacteria have been used for implant material infections. In this regard, Ag NPs have a substantial potential to replace with antibiotics due to their favorable antibacterial properties and broad antibacterial activity over other inorganic nanoparticles. Combining Ag NPs with implant materials for in vitro and in vivo studies makes them promising antibacterial implant materials. To reduce the toxicity of silver and to decrease the level of silver in the blood, future research should focus on developing effective techniques for combining silver nanoparticles with the implant materials that can release silver ions in a controlled and harmless way.

### Antibiotics

In addition to using inorganic antibacterial agents to prepare materials with antibacterial properties, antibiotics can also be used on titanium or its alloys’ surfaces to prepare nano-coatings or other NS to achieve antibacterial effects (Li et al., 2017a; Liu et al., 2017; Mohan Raj et al., 2018). The chosen antibiotics need to be able to kill Gram-positive cocci and Gram-positive bacillus effectively (Hickok and Shapiro, 2012). Available antibiotics include rifampicin, gentamicin, vancomycin, etc. (Simchi et al., 2011) (Table 6). Under ideal conditions, the antibiotics released by the prepared nanomaterials should reach the effective drug concentration and should maintain a long sufficient sterilization time (Salwiczek et al., 2014; Nguyen-Tri et al., 2019).

**TABLE 6:** The antibiotics used in various NS titanium and its alloys.

Materials	Antibiotic	Manufacturing technique	Base materials	Antibacterial effect/Antibacterial rate	Cells	Cytotoxicity	References
PCL-HA-Rf coating	Rifampicin	Electrospinning	Cp-Ti	*S. aureus*: >99.9%/24 h *MRSA*: >99.9%/24 h *S. epidermidis*: >99.9%/24 h *P. aeruginosa*: >99%/24 h	hFOB	No	Kranthi Kiran et al., 2019
HA-GS coating	Gentamicin	Vacuum cold spraying	Ti-6Al-4V	*E. coli*: 96.4%/31 days	Osteoblasts	No	Fathi et al., 2019
GS-PAA coating	Gentamicin	Layer-by-layer assembly	Ti-6Al-4V	*E. coli*: 99.93%/16 h *S. aureus*: 99.86%/16 h	–	–	He et al., 2020
Van-Sr-HAP nanocomposite	Vancomycin	Co-precipitation	Cp-Ti	*MRSA*: 99.45%/12 h *MSSA*: 98.22%/12 h	MSCs	No	David and Nallaiyan, 2018
NFX-PLGA coating	Norfloxacin	Nano spray drying	Cp-Ti	*E. coli*: 95.42∼99.93%/24 h	L929	No	Baghdan et al., 2019

## Bionic Nanostructures

In recent years, the wings of insects, such as dragonflies and cicadas, have attracted much attention as a model biological system because of their excellent antibacterial and antifungal properties (Ivanova et al., 2012; Diu et al., 2014; Kelleher et al., 2016). Some studies have shown the presence of physical nano-protrusions on the surface of insect wings. Their antibacterial properties may be due to the fact that when microbial cells come in contact with the protrusions, they possibility enhance the stress and deformation of the membrane structure of the microbial cells, leading to their destruction. It eventually leads to the dissolution and death of the cells (Ivanova et al., 2013; Nowlin et al., 2014; Bandara et al., 2017). By investigating the surface structure of insect wings as a model and preparing bionic structures according to it, new ideas for preparing modern antibacterial titanium alloys have emerged.

### Antibacterial Nanopatterns and Fabrication Methods

It has been shown that modification of the surface morphology of materials can be used to achieve antibacterial effects and inhibition of biofilm formation (Modaresifar et al., 2019; Stratakis et al., 2020). By changing the surface morphology without adding other chemical reagents, the antibacterial and antibiofilm formation properties can also be achieved (Campoccia et al., 2013; Hasan et al., 2013). Moreover, it has a slight effect on the mechanical strength of the material. Changing the surface morphology to prepare biomimetic structures with micron and nano-scale surface morphologies, and exploring the effect of surface morphology and size of titanium or its alloys that can effectively attack bacteria are the currently urgent problems to be solved. Table 7 lists some of the antibacterial NS formation methods and their properties.

**TABLE 7:** Antibacterial NS formation methods and the related properties.

Feature	Manufacturing technique	Base materials	Antibacterial effect and rate	Cells	Cytotoxicity	Wettability	Roughness	Morphology	References
Nanoflowers	Chemical etching + hydrothermal oxidation	Cp-Ti	*S. aureus*: 43.12%/24 h *MRSA*: 73.15%/24 h	hFOB	No	Hydrophilicity	Ra = 829 nm	A	Vishnu et al., 2019
Nanowires	Hydrothermal synthesis	Ti-6Al-4V	*S. aureus*: 74%/18 h	Osteoblasts	No	Hydrophilicity	–	B	Jaggessar et al., 2018
Regular nanotubes	Acid etching + anodic oxidation	Ti-6Al-4V	*E. coli*: 72.6%/2 h *S. aureus*: 68.2%/2 h	MG63	No	Hydrophilicity	Ra = 120 nm	C	Rahnamaee et al., 2020
Irregular nanotubes	Electrochemical anodization	Ti-6Al-4V	*E. coli*: 48.7%/2 h *S. aureus*: 50.8%/2 h	MG63	No	Superhydrophilicity	Ra = 360 nm	D	Rahnamaee et al., 2020
Nanotubes	Electrochemical anodization	Cp-Ti	*S. aureus*: 36.78%/16 h	–	–	Hydrophilicity	*R*_*rms*_ = 45.60 nm	E, F	Simi and Rajendran, 2017
Nano-ripples	Femtosecond laser direct writing	Cp-Ti	*E. coli*: 56%/24 h	MSCs	No	Superhydrophilicity	Ra = 274.6 nm	G	Luo et al., 2020
Nanoparticles	Aerosol flame synthesis	Aluminum alloy	*S. aureus*: 80%	–	–	Superhydrophilicity		H	De Falco et al., 2018
Nanopillars	Plasma etching	Cp-Ti	*P. aeruginosa*: 87 ± 2%/24 h *S. aureus*: 72.5 ± 13%/24 h	–	–	Hydrophobicity	–	I	Linklater et al., 2019

**FIGURE 6:**
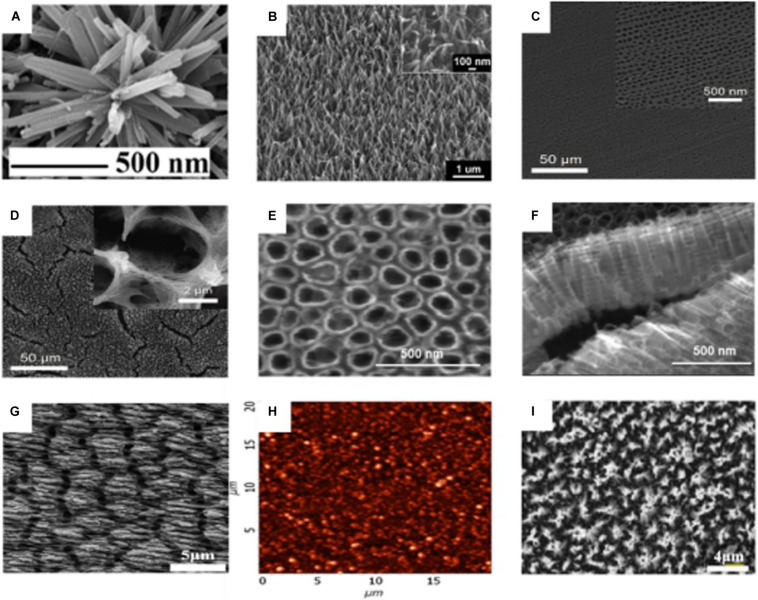
SEM micrograph of different nano-morphologies on the surface of titanium substrate. **(A)** Nanoflowers (Vishnu et al., 2019). **(B)** Nanowires (Jaggessar et al., 2018). **(C)** Regular nanotubes (Rahnamaee et al., 2020). **(D)** irregular nanotubes (Rahnamaee et al., 2020). **(E,F)** nanotubes (Simi and Rajendran, 2017). **(G)** Nano-ripples (Luo et al., 2020). **(H)** AFM micrograph of NPs (De Falco et al., 2018). **(I)** nanopillars (Linklater et al., 2019).

Different nano-morphologies can be prepared through different preparation processes: nanoflowers, nanowires, nanotubes, nano-ripples, NPs, and nanopillars are possible morphologies, Figure 6. Among these nano-topographies, nanopillars show good bactericidal effect, which may be related to its high aspect ratio (Linklater et al., 2018). In each nanotopography, cell activity was not significantly inhibited. Furthermore, in some nanotopographies, the cells’ metabolic activity tends to increase (Jaggessar et al., 2018).

**FIGURE 7:**
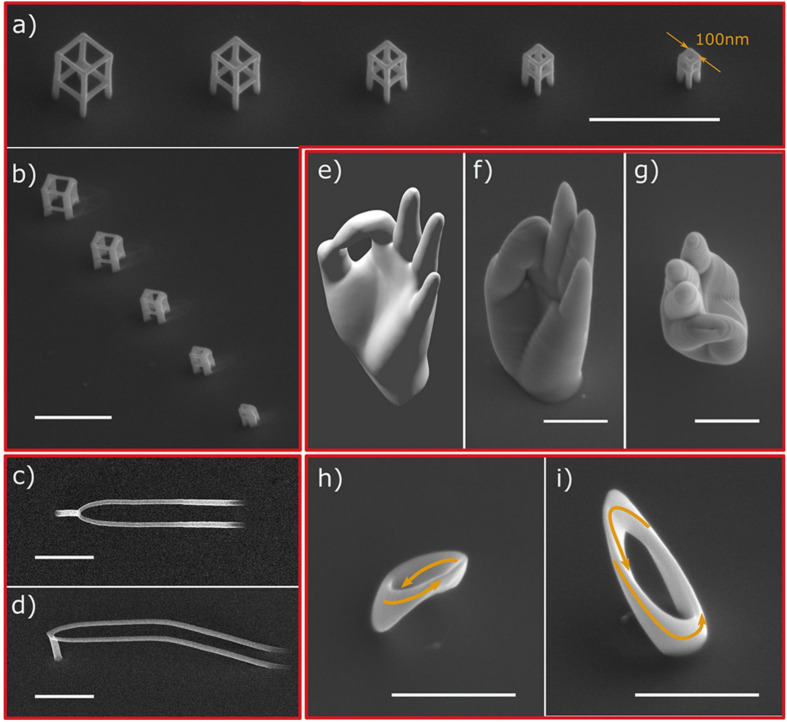
SEM images of different 3D geometries prepared by the focused electronbeam-induced deposition (FEBID) method. **(a,b)** A series of cubes. **(c,d)** Smooth nanowires. **(e–g)** Nanoscale replica of a human hand. **(h,i)** Cobalt Mobius strip (Skoric et al., 2020).

There are several methods to fabricate nanopatterns, and the commonly used methods are chemical etching (Heidarpour et al., 2020), reactive ion etching (Ganjian et al., 2019), plasma etching (He et al., 2013), hydrothermal synthesis (Jaggessar and Yarlagadda, 2020; Wategaonkar et al., 2020), and anodic oxidation (Mohan et al., 2020). The antibacterial nanopatterns with different antibacterial efficiencies have been prepared by these methods, but the antibacterial effect of these patterns is still not very satisfactory, which may be due to the fact that the production of antibacterial surfaces on titanium and its alloys are more difficult than other materials [silicon, polymethylmethacrylate (PMMA), etc.]. The development of nanopatterns with efficient antibacterial properties can enable the better clinical application of titanium-related medical materials and may address the bacterial resistance problem caused by antibiotic abuse. Currently, some novel methods for nanopatterning have been developed, such as two-photon polymerization (2PP) (Liao et al., 2020) and electron beam induced deposition (EBID) (Perentes et al., 2004; Skoric et al., 2020). Two-photon polymerization is a new 3D structure fabrication technology based on CAD/CAM, that precisely constructs 3D geometries with resolutions down to 100 nm. 2PP’s high resolution, adaptability to a wide range of materials, and the ability to create true 3D structures make it a very promising technology for the fabrication of medical implants (Doraiswamy et al., 2006; Ovsianikov and Chichkov, 2012). EBID fabrication technology is also gaining much attention (Hirt et al., 2017), enabling the fabrication of 3D structures in tens of nanometers and the deposition of a wide range of materials (metallic, organic, semiconducting, magnetic, superconducting, etc.) (Utke et al., 2008; Huth et al., 2018). EBID technology currently achieves vertical growth rates of hundreds of nanometers per second (Winkler et al., 2018) and can improve processing efficiency through several methods: optimizations of gas injection systems (GIS) (Friedli and Utke, 2009), deposition at low temperatures (Bresin et al., 2013), and simultaneous deposition of multiple beams (Riedesel et al., 2019). A variety of 3D geometries can be prepared by this method, as shown in Figure 7. In the future, the development of high-resolution, efficient, and controllable 3D nanomorphology methods will be a critical challenge to be overcome.

### Antibacterial Mechanism of Nanopatterns

Nanoscale structures with specific dimensions are fabricated on the surface of the substrate material, and this nanoscale structure performs mechanocidal action through different mechanisms. The interactions between nanopatterns and bacteria are multifaceted, and the exact mechanism of bactericidal action and the role of various factors in regulating bactericidal behavior are still controversial (Bandara et al., 2017; Linklater et al., 2017). Most researchers agree that nanopatterns with high aspect ratio are key factors in causing mechanical deformation of the bacterial cell walls, which in turn causes their rupture and death (Watson et al., 2015; Truong et al., 2017; Cao Y. et al., 2018). Xue et al. (2015) have developed mathematical models to explain the bactericidal properties of nanopatterns on the surface of cicada wings. Gravity as well as non-specific forces (such as van der Waals forces) have been demonstrated to play a role in bacterial cell wall rupture. In addition, extracellular polymeric substance (EPS) has been demonstrated to play an important role in regulating the bactericidal effects of nanopatterns, they are known as natural biopolymeric mixtures of proteins and polysaccharides which excreted by microorganisms. EPS plays an important role in the formation of biological membranes, also it promotes cell signaling and protects bacteria from the harmful effects of environmental factors (Limoli et al., 2015). Bandara et al. (2017) found that some bacteria under the influence of nanopillars secrete EPS with adhesion. Bacteria find the adherent surface unfit to survive and try to move away, and the anchoring action of EPS leads to rupture of the bacterial cell wall and bacterial death. Furthermore, the applied mechanical forces affect the metabolomics and bacterial genome and may also be the mechanism by which bacteria die on their surfaces (Rizzello et al., 2011; Belas, 2014; Persat, 2017; Velic et al., 2019). The mechanism of the antibacterial effect of nanopatterns is still not completely clarified, hence the research on the antibacterial mechanisms is crucial for the preparation of materials with excellent antibacterial effect.

### Factors Affecting the Antibacterial Property of Nanopatterns

The design parameters (height, diameter, and spacing) of the nanopatterns have a significant influence on the antibacterial properties. Nanopatterns with different parameters (height from 100 nm to 900 nm, diameter from 10 nm to 300 nm, and spacing less than 500 nm) have been reported in the literature to exhibit bactericidal properties (Modaresifar et al., 2019). Velic et al. (2019) studied the effect of varied design parameters of nanopatterns on bacteria behavior by developing a two-dimensional finite element model and demonstrated that reducing the pillar diameter could effectively promote bactericidal efficiency. Additionally, preparing nanopillar structures with similar diameters but different densities and heights showed that extra stretched nanopillars with varying heights leads to rupture in the bacterial cell membranes (the membranes exceeded the threshold value of stretching) (Wu et al., 2018).

The occurrence of implant material-related infections often begins with bacterial adhesion to the implant surface subsequently the colonization of bacteria and biofilm formation happens. Biofilms are bacteria produced microbial communities formed by the EPS substrate to inhibit the damage of bacteria. Statistically, about 99% of bacteria could exist with biofilm status (Zimmerli and Sendi, 2017). Biofilms exist as reservoirs for bacteria, often leading to chronic and systemic infections. Therefore, reducing the opportunity of bacteria to adhere to the surface of the implants is essential to prevent infection. The adhesion of bacteria to a material surface depends on the surface properties of the material, such as surface roughness, wettability, and topography. Bacteria are more likely to adhere to rough rather than smooth surfaces (Sarró et al., 2006; Fan et al., 2013) and more likely to adhere to hydrophobic rather than hydrophilic surfaces (Pagedar et al., 2010). At the same time, the nanoscale surface has a higher resistance to bacterial adhesion than the micron and macroscale surfaces (Singh et al., 2011; Spengler et al., 2019). The severe plastic deformation (SPD) process is a recently developed technique for the fabrication of nanostructures (Dyakonov et al., 2019; Shuitcev et al., 2020). It is noteworthy that by reducing the grain size to the sub-micron range (100–500 nm) through SPD processes, the mechanical properties of commercially pure titanium are comparable to those of conventional Ti-6Al-4V alloys (Singh et al., 2011). It was found that the increase in Ra of the pure titanium surface after SPD processing was followed by an increase in the adhesion density of *S. aureus*, which was independent to *P. aeruginosa*. The effect of surface hydrophilicity on bacterial adhesion can be explained by thermodynamics (Bruinsma et al., 2001), with a higher affinity of hydrophobic bacteria for hydrophobic surfaces and of hydrophilic bacteria for hydrophilic surfaces (Sabirov et al., 2015).

## Conclusion

Nanomaterials have the characteristics of small size and large specific surface area which lead them to have more potent antibacterial activity and drug loading capacity than traditional materials, thus showing an excellent antibacterial effect. However, once the safe antimicrobial material exceeds its safe dose during the release process, it will produce cellular cytotoxicity and even affect the osteogenic performance of titanium implant materials’ surfaces. Moreover, the antibacterial ability of the implant material surface will gradually weaken with the release of antibacterial substances. The nano-morphology on the surface of the titanium implant material can achieve a long-term antibacterial effect. However, the destructive effect of the nano-morphology surface on different bacteria is quite different, and the antibacterial range is limited. At present, the nanometer modification of implant materials is still in its infancy and lots of improvement must be done in order to make them an ideal option for medical applications both form cytotoxicity and functionality, this study aims to produce a guideline and achieve the as-mentioned goals. In this regard, nanostructure formation methods including template synthesis, electrochemical anodization, and hydrothermal treatment was discussed. In addition, nano coating methods such as CVD, PVD, sol-gel, spin coating, electrophoretic deposition, plasma spraying, and LBL technologies were introduced. Then different antibacterial agent loading on these nanostructured surfaces were analyzed. The loading substances can have metallic nature like Ag, Cu, Zn, Au, and Ni, even some antibiotics including rifampicin, gentamicin, and vancomycin can be loaded on these nanostructures.

Although, there are many known involved parameters and methods, it seems that more research in this field is from crucial importance since it affect the health issues. Some modern, safe and economic methods and materials should be introduced by researchers. The accurate and complementary guidelines about the safe thresholds and stability of antibacterial agents and procedures must be prepared. The precise mechanisms both from bactericidal and functionality points of view should be understood. The effect of antibacterial schemes on biocompatibility, osteogenic activity, genotoxicity, and their possible influence on periphery tissues should be analyzed. In future, the direction of research shifts to improve the antibacterial properties of non-cytotoxic antibacterial nanoparticles and develop implant materials with stable and long-lasting antibacterial effects. In the study of cytotoxic nanoparticles with antibacterial properties (e.g., silver nanoparticles), further clarifying the factors and mechanisms leading to cytotoxicity and developing controllable and harmless antibacterial materials are the next research focuses. The possible bactericidal mechanisms, the design parameters to achieve the efficient antibacterial performance on different bacteria species, and the development of high-precision nanopattern processing technologies are still among the future problems to be solved in nanopattern antibacterial studies. This review paper can help the investigators to develop the new methods, procedures, and materials to attain a modern scheme in design of nanostructured antibacterial materials with long-last, safe, biocompatible, and effective on broad bacterial infections characteristics.

## Author Contributions

JQL, JL, and SA contributed equally to the design, reorganize the figures, and writing the manuscript. CW, MT, KX, and CY collated the resource. JY, LW, CL, and YT helped with editing the manuscript. YT conceived and designed the outline of this review. All the authors read and approved the final manuscript.

## Conflict of Interest

The authors declare that the research was conducted in the absence of any commercial or financial relationships that could be construed as a potential conflict of interest.

## References

[B1] Aal N. A. Al-Hazmi F. Al-Ghamdi A. A. Al-Ghamdi A. A. El-Tantawy F. Yakuphanoglu F. (2015). Novel rapid synthesis of zinc oxide nanotubes via hydrothermal technique and antibacterial properties. *Spectrochim. Acta Part A Mol. Biomol. Spectrosc.* 135 871–877. 10.1016/j.saa.2014.07.099 25155943

[B2] Abid S. A. Taha A. A. Ismail R. A. Mohsin M. H. (2020). Antibacterial and cytotoxic activities of cerium oxide nanoparticles prepared by laser ablation in liquid. *Environ. Sci. Pollut. Res.* 27 30479–30489. 10.1007/s11356-020-09332-9 32468358

[B3] Abu-Thabit N. Y. Makhlouf A. S. H. (2020). *Fundamental of Smart Coatings and Thin Films: Synthesis, Deposition Methods, and Industrial Applications.* (Amsterdam: Elsevier Inc). 10.1016/b978-0-12-849870-5.00001-x

[B4] Ahmed K. B. A. Raman T. Veerappan A. (2016). Future prospects of antibacterial metal nanoparticles as enzyme inhibitor. *Mater. Sci. Eng. C* 68 939–947. 10.1016/j.msec.2016.06.034 27524096

[B5] Akhtar M. A. Ilyas K. Siska F. Boccaccini A. R. (2020). Electrophoretic deposition of copper(II)-chitosan complexes for antibacterial coatings. *Int. J. Mol. Sci.* 21:2637. 10.3390/ijms21072637 32290155 PMC7177350

[B6] Akram W. Rafique A. F. Maqsood N. Khan A. Badshah S. Khan R. U. (2020). Characterization of PTFE film on 316L stainless steel deposited through spin coating and its anticorrosion performance in multi acidic mediums. *Materials* 13:388. 10.3390/ma13020388 31947700 PMC7014069

[B7] Alaei M. Atapour M. Labbaf S. (2020). Electrophoretic deposition of chitosan-bioactive glass nanocomposite coatings on AZ91 Mg alloy for biomedical applications. *Prog. Org. Coatings* 147:105803 10.1016/j.porgcoat.2020.105803

[B8] Alajlani Y. Placido F. Barlow A. Chu H. O. Song S. Ur Rahman S. (2017). Characterisation of Cu2O, Cu4O3, and CuO mixed phase thin films produced by microwave-activated reactive sputtering. *Vacuum* 144 217–228. 10.1016/j.vacuum.2017.08.005

[B9] Alajlani Y. Placido F. Gibson D. Chu H. O. Song S. Porteous L. (2016). Nanostructured ZnO films prepared by hydro-thermal chemical deposition and microwave-activated reactive sputtering. *Surf. Coatings Technol.* 290 16–20. 10.1016/j.surfcoat.2016.01.036

[B10] Al-Douri Y. Fakhri M. A. Badi N. Voon C. H. (2018). Effect of stirring time on the structural parameters of nanophotonic LiNbO3 deposited by spin-coating technique. *Optik* 156 886–890. 10.1016/j.ijleo.2017.12.059

[B11] Alivov Y. Pandikunta M. Nikishin S. Fan Z. Y. (2009). The anodization voltage influence on the properties of TiO2 nanotubes grown by electrochemical oxidation. *Nanotechnology* 20:225602 10.1088/0957-4484/20/22/225602 19436088

[B12] Andersson D. I. Hughes D. (2012). Evolution of antibiotic resistance at non-lethal drug concentrations. *Drug Resist. Updat.* 15 162–172. 10.1016/j.drup.2012.03.005 22516308

[B13] Andersson D. I. Hughes D. (2014). Microbiological effects of sublethal levels of antibiotics. *Nat. Rev. Microbiol.* 12 465–478. 10.1038/nrmicro3270 24861036

[B14] Anitha S. Muthukumaran S. (2020). Structural, optical and antibacterial investigation of La, Cu dual doped ZnO nanoparticles prepared by co-precipitation method. *Mater. Sci. Eng. C. Mater. Biol. Appl.* 108:110387. 10.1016/j.msec.2019.110387 31924039

[B15] Antonelli D. M. Ying J. Y. (1995). Synthesis of Hexagonally Packed Mesoporous TiO2 by a Modified Sol–Gel Method. *Angew. Chem. Int. Ed. Engl.* 34 2014–2017. 10.1002/anie.199520141

[B16] AshaRani P. V. Low Kah, Mun G. Hande M. P. Valiyaveettil S. (2009). Cytotoxicity and genotoxicity of silver nanoparticles in human cells. *ACS Nano* 3 279–290. 10.1021/nn800596w 19236062

[B17] Attarilar S. Salehi M. T. Al-Fadhalah K. J. Djavanroodi F. Mozafari M. (2019a). Functionally graded titanium implants: characteristic enhancement induced by combined severe plastic deformation. *PLoS One* 14:e0221491. 10.1371/journal.pone.0221491 31442256 PMC6707610

[B18] Attarilar S. Salehi M.-T. Djavanroodi F. (2019b). Microhardness evolution of pure titanium deformed by equal channel angular extrusion. *Metall. Res. Technol.* 116:408 10.1051/metal/2018135

[B19] Attarilar S. Yang J. Ebrahimi M. Wang Q. Liu J. Tang Y. (2020). The toxicity phenomenon and the related occurrence in metal and metal oxide nanoparticles: a brief review from the biomedical perspective. *Front. Bioeng. Biotechnol.* 8:822. 10.3389/fbioe.2020.00822 32766232 PMC7380248

[B20] Avcu E. Baştan F. E. Abdullah H. Z. Rehman M. A. U. Avcu Y. Y. Boccaccini A. R. (2019). Electrophoretic deposition of chitosan-based composite coatings for biomedical applications: a review. *Prog. Mater. Sci.* 103 69–108. 10.1016/j.pmatsci.2019.01.001

[B21] Avila J. D. Bose S. Bandyopadhyay A. (2018). “Additive manufacturing of titanium and titanium alloys for biomedical applications,” in *Additive Manufacturing of Emerging Materials*, eds F. Froes and M. Qian (Amsterdam: Elsevier Inc), 325–343. 10.1016/B978-0-12-812456-7.00015-9

[B22] Avino F. Fonnesu D. Koettig T. Bonura M. Senatore C. Perez Fontenla A. T. (2020). Improved film density for coatings at grazing angle of incidence in high power impulse magnetron sputtering with positive pulse. *Thin Solid Films* 706:138058 10.1016/j.tsf.2020.138058

[B23] Baghdan E. Raschpichler M. Lutfi W. Pinnapireddy S. R. Pourasghar M. Schäfer J. (2019). Nano spray dried antibacterial coatings for dental implants. *Eur. J. Pharm. Biopharm.* 139 59–67. 10.1016/j.ejpb.2019.03.003 30836179

[B24] Bakhshandeh S. Amin Yavari S. (2018). Electrophoretic deposition: a versatile tool against biomaterial associated infections. *J. Mater. Chem. B* 6 1128–1148. 10.1039/c7tb02445b 32254176

[B25] Bandara C. D. Singh S. Afara I. O. Wolff A. Tesfamichael T. Ostrikov K. (2017). Bactericidal effects of natural nanotopography of dragonfly wing on *Escherichia coli*. *ACS Appl. Mater. Interfaces* 9 6746–6760. 10.1021/acsami.6b13666 28139904

[B26] Belas R. (2014). Biofilms, flagella, and mechanosensing of surfaces by bacteria. *Trends Microbiol.* 22 517–527. 10.1016/j.tim.2014.05.002 24894628

[B27] Ben-Knaz Wakshlak R. Pedahzur R. Menagen B. Avnir D. (2016). An antibacterial copper composite more bioactive than metallic silver. *J. Mater. Chem. B* 4 4322–4329. 10.1039/c6tb00719h 32263414

[B28] Bera D. Kuiry S. C. Seal S. (2004). Synthesis of nanostructured materials using template-assisted Electrodeposition. *JOM* 56 49–53. 10.1007/s11837-004-0273-5

[B29] Bernal A. Tselev A. Kalinin S. Bassiri-Gharb N. (2012). Free-standing ferroelectric nanotubes processed via soft-template infiltration. *Adv. Mater.* 24 1160–1165. 10.1002/adma.201103993 22279013

[B30] Besra L. Liu M. (2007). A review on fundamentals and applications of electrophoretic deposition (EPD). *Prog. Mater. Sci.* 52 1–61. 10.1016/j.pmatsci.2006.07.001

[B31] Bhadra C. U. Jonas Davidson D. Henry Raja D. (2020). Fabrication of titanium oxide nanotubes by varying the anodization time. *Mater. Today Proc.* 10.1016/j.matpr.2020.01.455

[B32] Biju K. P. Subrahmanyam A. Jain M. K. (2009). Growth of InN nanocrystalline films by activated reactive evaporation. *J. Nanosci. Nanotechnol.* 9 5208–5213. 10.1166/jnn.2009.1123 19928202

[B33] Boccaccini A. R. Cho J. Subhani T. Kaya C. Kaya F. (2010). Electrophoretic deposition of carbon nanotube-ceramic nanocomposites. *J. Eur. Ceram. Soc.* 30 1115–1129. 10.1016/j.jeurceramsoc.2009.03.016

[B34] Bozkurt Çırak B Karadeniz S. M. Kılınç T. Caglar B. Ekinci A. E. Yelgin H. (2017). Synthesis, surface properties, crystal structure and dye sensitized solar cell performance of TiO2 nanotube arrays anodized under different voltages. *Vacuum* 144 183–189. 10.1016/j.vacuum.2017.07.037

[B35] Braem A. De Brucker K. Delattin N. Killian M. S. Roeffaers M. B. J. Yoshioka T. (2017). Alternating current electrophoretic deposition for the immobilization of antimicrobial agents on titanium implant surfaces. *ACS Appl. Mater. Interfaces* 9 8533–8546. 10.1021/acsami.6b16433 28211996

[B36] Bresin M. Toth M. Dunn K. A. (2013). Direct-write 3D nanolithography at cryogenic temperatures. *Nanotechnology* 24:035301 10.1088/0957-4484/24/3/035301 23263276

[B37] Bruinsma G. M. Rustema-Abbing M. Van Der Mei H. C. Busscher H. J. (2001). Effects of cell surface damage on surface properties and adhesion of *Pseudomonas aeruginosa*. *J. Microbiol. Methods* 45 95–101. 10.1016/S0167-7012(01)00238-X 11311394

[B38] Bulla D. A. P. Li W. T. Charles C. Boswell R. Ankiewicz A. Love J. (2004). Deposition and characterization of silica-based films by helicon-activated reactive evaporation applied to optical waveguide fabrication. *Appl. Opt.* 43 2978–2985. 10.1364/AO.43.002978 15143826

[B39] Buzea C. Pacheco I. (2017). Nanomaterials and their classification. *Adv. Struct. Mater.* 62 3–45. 10.1007/978-81-322-3655-9_1

[B40] Campoccia D. Montanaro L. Arciola C. R. (2013). A review of the biomaterials technologies for infection-resistant surfaces. *Biomaterials* 34 8533–8554. 10.1016/j.biomaterials.2013.07.089 23953781

[B41] Cao M. Zhao W. Wang L. Li R. Gong H. Zhang Y. (2018). Graphene oxide-assisted accumulation and layer-by-layer assembly of antibacterial peptide for sustained release applications. *ACS Appl. Mater. Interfaces* 10 24937–24946. 10.1021/acsami.8b07417 29956912

[B42] Cao S. Huang W. Wu L. Tian M. Song Y. (2018). On the interfacial adhesion between TiO2 nanotube array Layer and Ti Substrate. *Langmuir* 34 13888–13896. 10.1021/acs.langmuir.8b03408 30362766

[B43] Cao Y. Su B. Chinnaraj S. Jana S. Bowen L. Charlton S. (2018). Nanostructured titanium surfaces exhibit recalcitrance towards Staphylococcus epidermidis biofilm formation. *Sci. Rep.* 8:1071. 10.1038/s41598-018-19484-x 29348582 PMC5773551

[B44] Cheang P. Khor K. A. (1996). Addressing processing problems associated with plasma spraying of hydroxyapatite coatings. *Biomaterials* 17 537–544. 10.1016/0142-9612(96)82729-3 8991486

[B45] Chen B. You Y. Ma A. Song Y. Jiao J. Song L. (2020). Zn-Incorporated TiO2 nanotube surface improves osteogenesis ability through influencing immunomodulatory function of macrophages. *Int. J. Nanomed.* 15 2095–2118. 10.2147/IJN.S244349 PMC7109325 32273705

[B46] Chen J. Wang F. Liu Q. Du J. (2014). Antibacterial polymeric nanostructures for biomedical applications. *Chem. Commun.* 50 14482–14493. 10.1039/C4CC03001J 25110921

[B47] Chen Y. Gao A. Bai L. Wang Y. Wang X. Zhang X. (2017). Antibacterial, osteogenic, and angiogenic activities of SrTiO3 nanotubes embedded with Ag2O nanoparticles. *Mater. Sci. Eng. C* 75 1049–1058. 10.1016/j.msec.2017.03.014 28415389

[B48] Cheng Y. F. Zhang J. Y. Wang Y. B. Li C. M. Lu Z. S. Hu X. F. (2019). Deposition of catechol-functionalized chitosan and silver nanoparticles on biomedical titanium surfaces for antibacterial application. *Mater. Sci. Eng. C* 98 649–656. 10.1016/j.msec.2019.01.019 30813068

[B49] Chi B. Victorio E. S. Jin T. (2007). Synthesis of TiO 2-based nanotube on Ti substrate by hydrothermal treatment. *J. Nanosci. Nanotechnol.* 7 668–672. 10.1166/jnn.2007.147 17450812

[B50] Choi D. S. Kim C. Lim J. Cho S. H. Lee G. Y. Lee H. J. (2018). Ultrastable graphene-encapsulated 3 nm nanoparticles by in situ chemical vapor deposition. *Adv. Mater.* 30:1805023. 10.1002/adma.201805023 30318636

[B51] Choi H. Kim J.-J. Mo Y.-H. Reddy B. M. Park S.-E. (2017). Novelty of dynamic process in the synthesis of biocompatible silica nanotubes by biomimetic glycyldodecylamide as a soft template. *Langmuir* 33 10707–10714. 10.1021/acs.langmuir.7b02881 28920442

[B52] Chouirfa H. Bouloussa H. Migonney V. Falentin-Daudre C. (2019). Review of titanium surface modification techniques and coatings for antibacterial applications. *Acta Biomater.* 83 37–54. 10.1016/j.actbio.2018.10.036 30541702

[B53] Chtouki T. Soumahoro L. Kulyk B. Bougharraf H. Erguig H. Ammous K. (2017). Comparative study on the structural, morphological, linear and nonlinear optical properties of CZTS thin films prepared by spin-coating and spray pyrolysis. *Mater. Today Proc.* 4 5146–5153. 10.1016/j.matpr.2017.05.020

[B54] Cipriano A. F. Miller C. Liu H. (2014). Anodic growth and biomedical applications of TiO2 nanotubes. *J. Biomed. Nanotechnol.* 10 2977–3003. 10.1166/jbn.2014.1927 25992426

[B55] Cui C. Cui X. Li X. Luo K. Lu J. Ren X. (2018). Plastic-deformation-driven SiC nanoparticle implantation in an Al surface by laser shock wave: mechanical properties, microstructure characteristics, and synergistic strengthening mechanisms. *Int. J. Plast.* 102 83–100. 10.1016/j.ijplas.2017.12.004

[B56] David N. Nallaiyan R. (2018). Biologically anchored chitosan/gelatin-SrHAP scaffold fabricated on Titanium against chronic osteomyelitis infection. *Int. J. Biol. Macromol.* 110 206–214. 10.1016/j.ijbiomac.2017.11.174 29197567

[B57] De Falco G. Ciardiello R. Commodo M. Del Gaudio P. Minutolo P. Porta A. (2018). TIO2 nanoparticle coatings with advanced antibacterial and hydrophilic properties prepared by flame aerosol synthesis and thermophoretic deposition. *Surf. Coatings Technol.* 349 830–837. 10.1016/j.surfcoat.2018.06.083

[B58] Delfini A. Vricella A. Morles R. B. Pastore R. Micheli D. Gugliermetti F. (2017). CVD nano-coating of carbon composites for space materials atomic oxygen shielding. *Procedia Struct. Integr.* 3 208–216. 10.1016/j.prostr.2017.04.047

[B59] Deng K. Luo Z. Tan L. Quan Z. (2020). Self-assembly of anisotropic nanoparticles into functional superstructures. *Chem. Soc. Rev.* 49 6002–6038. 10.1039/d0cs00541j 32692337

[B60] Deshmukh S. P. Mullani S. B. Koli V. B. Patil S. M. Kasabe P. J. Dandge P. B. (2018). Ag Nanoparticles Connected to the Surface of TiO2 Electrostatically for Antibacterial Photoinactivation Studies. *Photochem. Photobiol.* 94 1249–1262. 10.1111/php.12983 30025150

[B61] Diu T. Faruqui N. Sjöström T. Lamarre B. Jenkinson H. F. Su B. (2014). Cicada-inspired cell-instructive nanopatterned arrays. *Sci. Rep.* 4:7122. 10.1038/srep07122 25409910 PMC4238011

[B62] Doraiswamy A. Jin C. Narayan R. J. Mageswaran P. Mente P. Modi R. (2006). Two photon induced polymerization of organic-inorganic hybrid biomaterials for microstructured medical devices. *Acta Biomater.* 2 267–275. 10.1016/j.actbio.2006.01.004 16701886

[B63] Dumitrache F. Morjan I. P. Dutu E. Fleaca C. T. Scarisoreanu M. (2019). Zn/F-doped tin oxide nanoparticles synthesized by laser pyrolysis: structural and optical properties. *Beilstein J. Nanotechnol.* 10 9–21. 10.3762/bjnano.10.2 30680275 PMC6334807

[B64] Dyakonov G. S. Mironov S. Semenova I. P. Valiev R. Z. Semiatin S. L. (2017). Microstructure evolution and strengthening mechanisms in commercial-purity titanium subjected to equal-channel angular pressing. *Mater. Sci. Eng. A* 701 289–301. 10.1016/j.msea.2017.06.079

[B65] Dyakonov G. S. Mironov S. Semenova I. P. Valiev R. Z. Semiatin S. L. (2019). EBSD analysis of grain-refinement mechanisms operating during equal-channel angular pressing of commercial-purity titanium. *Acta Mater.* 173 174–183. 10.1016/j.actamat.2019.05.014

[B66] Elbourne A. Chapman J. Gelmi A. Cozzolino D. Crawford R. J. Truong V. K. (2019). Bacterial-nanostructure interactions: the role of cell elasticity and adhesion forces. *J. Colloid Interface Sci.* 546 192–210. 10.1016/j.jcis.2019.03.050 30921674

[B67] Elmi F. Valipour E. Ghasemi S. (2019). Synthesis of anticorrosion nanohybrid films based on bioinspired dopamine, L-cys/CNT@PDA through self-assembly on 304 stainless steel in 3.5*% NaCl*. *Bioelectrochemistry* 126 79–85. 10.1016/j.bioelechem.2018.11.012 30530055

[B68] Endrino J. L. Nainaparampil J. J. Krzanowski E. (2002). Microstructure and vacuum tribology of TiC–Ag composite coatings deposited by magnetron sputtering-pulsed laser deposition.pdf. *Surf. Coatings Technol.* 157 95–101.

[B69] Eom T. Woo K. Cho W. Heo J. E. Jang D. Shin J. I. (2017). Nanoarchitecturing of natural melanin nanospheres by layer-by-layer assembly: macroscale anti-inflammatory conductive coatings with optoelectronic tunability. *Biomacromolecules* 18 1908–1917. 10.1021/acs.biomac.7b00336 28510430

[B70] Fan A. Zhang H. Ma Y. Zhang X. Zhang J. Tang B. (2013). Bacteria adherence properties of nitrided layer on Ti6Al4V by the plasma nitriding technique. *J. Wuhan Univ. Technol. Mater. Sci. Ed.* 28 1223–1226. 10.1007/s11595-013-0849-4

[B71] Fathi M. Akbari B. Taheriazam A. (2019). Antibiotics drug release controlling and osteoblast adhesion from Titania nanotubes arrays using silk fibroin coating. *Mater. Sci. Eng. C* 103:109743. 10.1016/j.msec.2019.109743 31349530

[B72] Fathy Fahim N. Sekino T. Farouk Morks M. Kusunose T. (2009). Electrochemical growth of vertically-oriented high aspect ratio titania nanotubes by rabid anodization in fluoride-free media. *J. Nanosci. Nanotechnol.* 9 1803–1818. 10.1166/jnn.2009.440 19435043

[B73] Fauchais P. Montavon G. (2007). Plasma spraying: from plasma generation to coating structure. *Adv. Heat Transf.* 40 205–344. 10.1016/S0065-2717(07)40003-X

[B74] Fernandes E. G. R. Brazaca L. C. Rodríguez-Mendez M. L. de Saja J. A. Zucolotto V. (2011). Immobilization of lutetium bisphthalocyanine in nanostructured biomimetic sensors using the LbL technique for phenol detection. *Biosens. Bioelectron.* 26 4715–4719. 10.1016/j.bios.2011.05.032 21704506

[B75] Fernández-Arias M. Boutinguiza M. del Val J. Riveiro A. Rodríguez D. Arias-González F. (2020). Fabrication and deposition of copper and copper oxide nanoparticles by laser ablation in open air. *Nanomaterials* 10:300. 10.3390/nano10020300 32050620 PMC7075210

[B76] Foldbjerg R. Jiang X. Micluş T. Chen C. Autrup H. Beer C. (2015). Silver nanoparticles - Wolves in sheep’s clothing? *Toxicol. Res.* 4 563–575. 10.1039/c4tx00110a

[B77] Friedli V. Utke I. (2009). Optimized molecule supply from nozzle-based gas injection systems for focused electron- and ion-beam induced deposition and etching: simulation and experiment. *J. Phys. D Appl. Phys.* 42:125305 10.1088/0022-3727/42/12/125305

[B78] Fu X. Cai J. Zhang X. Li W. Di, Ge H. (2018). Top-down fabrication of shape-controlled, monodisperse nanoparticles for biomedical applications. *Adv. Drug Deliv. Rev.* 132 169–187. 10.1016/j.addr.2018.07.006 30009884

[B79] Ganjian M. Modaresifar K. Zhang H. Hagedoorn P. L. Fratila-Apachitei L. E. Zadpoor A. A. (2019). Reactive ion etching for fabrication of biofunctional titanium nanostructures. *Sci. Rep.* 9:36857. 10.1038/s41598-019-55093-y 31827149 PMC6906493

[B80] Gao C. Cheng H. Xu N. Li Y. Chen Y. Wei Y. (2019). Poly(dopamine) and Ag nanoparticle-loaded TiO 2 nanotubes with optimized antibacterial and ROS-scavenging bioactivities. *Nanomedicine* 14 803–818. 10.2217/nnm-2018-0131 30638128

[B81] Gao Z. L. Zhang K. Yuen M. M. F. (2011). Fabrication of carbon nanotube thermal interface material on aluminum alloy substrates with low pressure CVD. *Nanotechnology* 22:265611 10.1088/0957-4484/22/26/265611 21576791

[B82] Garbacz H. Wieciński P. Adamczyk-Cieślak B. Mizera J. Kurzydłowski K. J. (2010). Studies of aluminium coatings deposited by vacuum evaporation and magnetron sputtering. *J. Microsc.* 237 475–480. 10.1111/j.1365-2818.2009.03297.x 20500421

[B83] Geng Z. Wang R. Zhuo X. Li Z. Huang Y.-C. Ma L. (2016). Incorporation of silver and strontium in hydroxyapatite coating on titanium surface for enhanced antibacterial and biological properties. *Mater. Sci. Eng. C* 71 852–861. 10.1016/j.msec.2016.10.079 27987782

[B84] Ghalayani Esfahani A. Soleimanzade M. Campiglio C. E. Federici A. Altomare L. Draghi L. (2019). Hierarchical microchannel architecture in chitosan/bioactive glass scaffolds via electrophoretic deposition positive-replica. *J. Biomed. Mater. Res. Part A* 107 1455–1465. 10.1002/jbm.a.36660 30786159

[B85] Gleiter H. (2000). Nanostructured materials: basic concepts and microstructure. *Acta Mater.* 48 1–29. 10.1016/S1359-6454(99)00285-2

[B86] Gode C. Attarilar S. Eghbali B. Ebrahimi M. (2015). Electrochemical behavior of equal channel angular pressed titanium for biomedical application. *AIP Conf. Proc.* 1653:020041 10.1063/1.4914232

[B87] Gokcekaya O. Webster T. Ueda K. Narushima T. Ergun C. (2017). *In vitro* performance of Ag-incorporated hydroxyapatite and its adhesive porous coatings deposited by electrostatic spraying. *Mater. Sci. Eng. C* 77 556–564. 10.1016/j.msec.2017.03.233 28532065

[B88] Gonçalves M. C. (2018). Sol-gel silica nanoparticles in medicine: a natural choice. design, synthesis and products. *Molecules* 23:2021. 10.3390/molecules23082021 30104542 PMC6222648

[B89] Gong D. Grimes C. A. Varghese O. K. Hu W. Singh R. S. Chen Z. (2001). Titanium oxide nanotube arrays prepared by anodic oxidation. *J. Mater. Res.* 16 3331–3334. 10.1557/JMR.2001.0457

[B90] Gong Y. Tu R. Goto T. (2013). High-speed deposition of titanium carbide coatings by laser-assisted metal-organic CVD. *Mater. Res. Bull.* 48 2766–2770. 10.1016/j.materresbull.2013.03.039

[B91] Goudarzi M. Saviz S. Ghoranneviss M. Salar Elahi A. (2018). Antibacterial characteristics of thermal plasma spray system. *J. Xray Sci. Technol.* 26 509–521. 10.3233/XST-17318 29562572

[B92] Gour A. Jain N. K. (2019). Advances in green synthesis of nanoparticles. *Artif. Cells Nanomed. Biotechnol.* 47 844–851. 10.1080/21691401.2019.1577878 30879351

[B93] Grandi S. Cassinelli V. Bini M. Saino E. Mustarelli P. Arciola C. R. (2011). Bone Reconstruction: Au Nanocomposite Bioglasses with Antibacterial Properties. *Int. J. Artif. Organs* 34 920–928. 10.5301/ijao.5000059 22094575

[B94] Guan M. Chen Y. Wei Y. Song H. Gao C. Cheng H. (2019). Long-lasting bactericidal activity through selective physical puncture and controlled ions release of polydopamine and silver nanoparticles-loaded TiO2 nanorods *in vitro* and *in vivo*. *Int. J. Nanomed.* 14 2903–2914. 10.2147/IJN.S202625 31114199 PMC6497113

[B95] Gunputh U. F. Le H. Handy R. D. Tredwin C. (2018). Anodised TiO2 nanotubes as a scaffold for antibacterial silver nanoparticles on titanium implants. *Mater. Sci. Eng. C* 91 638–644. 10.1016/j.msec.2018.05.074 30033297

[B96] Guo Y. Chen D. Cheng M. Lu W. Wang L. Zhang X. (2013). The bone tissue compatibility of a new Ti35Nb2Ta3Zr alloy with a low Young’s modulus. *Int. J. Mol. Med.* 31 689–697. 10.3892/ijmm.2013.1249 23338484

[B97] Guo Z. Chen Y. Wang Y. Jiang H. Wang X. (2020). Advances and challenges in metallic nanomaterial synthesis and antibacterial applications. *J. Mater. Chem. B* 8 4764–4777. 10.1039/d0tb00099j 32207511

[B98] Gupta A. Srivastava R. (2019). Mini submersible pump assisted sonochemical reactors: large-scale synthesis of zinc oxide nanoparticles and nanoleaves for antibacterial and anti-counterfeiting applications. *Ultrason. Sonochem.* 52 414–427. 10.1016/j.ultsonch.2018.12.020 30755387

[B99] Gupta M. C. Ungaro C. Foley J. J. Gray S. K. (2018). Optical nanostructures design, fabrication, and applications for solar/thermal energy conversion. *Sol. Energy* 165, 100–114. 10.1016/j.solener.2018.01.010

[B100] Gutés A. Carraro C. Maboudian R. (2012). Single-layer CVD-grown graphene decorated with metal nanoparticles as a promising biosensing platform. *Biosens. Bioelectron.* 33 56–59. 10.1016/j.bios.2011.12.018 22240266

[B101] Hadidi M. Bigham A. Saebnoori E. Hassanzadeh-Tabrizi S. A. Rahmati S. Alizadeh Z. M. (2017). Electrophoretic-deposited hydroxyapatite-copper nanocomposite as an antibacterial coating for biomedical applications. *Surf. Coatings Technol.* 321 171–179. 10.1016/j.surfcoat.2017.04.055

[B102] Hafeez N. Liu J. Wang L. Wei D. Tang Y. Lu W. (2020). Superelastic response of low-modulus porous beta-type Ti-35Nb-2Ta-3Zr alloy fabricated by laser powder bed fusion. *Addit. Manuf.* 34:101264 10.1016/j.addma.2020.101264

[B103] Hafeez N. Liu S. Lu E. Wang L. Liu R. Lu W. (2019). Mechanical behavior and phase transformation of β-type Ti-35Nb-2Ta-3Zr alloy fabricated by 3D-Printing. *J. Alloys Compd.* 790 117–126. 10.1016/j.jallcom.2019.03.138

[B104] Hameed P. Gopal V. Bjorklund S. Ganvir A. Sen D. Markocsan N. (2019). Axial Suspension Plasma Spraying: an ultimate technique to tailor Ti6Al4V surface with HAp for orthopaedic applications. *Colloids Surfaces B Biointerfaces* 173 806–815. 10.1016/j.colsurfb.2018.10.071 30551296

[B105] Hanawa T. (2018). *Transition of Surface Modification of Titanium for Medical and Dental Use.* Amsterdam: Elsevier Inc 10.1016/B978-0-12-812456-7.00005-6

[B106] Hang R. Liu Y. Bai L. Zhang X. Huang X. Jia H. (2018). Length-dependent corrosion behavior, Ni(2+) release, cytocompatibility, and antibacterial ability of Ni-Ti-O nanopores anodically grown on biomedical NiTi alloy. *Mater. Sci. Eng. C Mater. Biol. Appl.* 89 1–7. 10.1016/j.msec.2018.03.018 29752078

[B107] Hang R. Zhang M. Ma S. Chu P. (2012). Biological response of endothelial cells to diamond-like carbon-coated NiTi alloy. *J. Biomed. Mater. Res. A* 100 496–506. 10.1002/jbm.a.33295 22125203

[B108] Haque A. K. M. M. Kim S. Kim J. Noh J. Huh S. Choi B. (2018). Surface modification of graphene nanoparticles by acid treatment and grinding process. *J. Nanosci. Nanotechnol.* 18 645–650. 10.1166/jnn.2018.13928 29768890

[B109] Harsha N. Ranya K. R. Babitha K. B. Shukla S. Biju S. Reddy M. L. P. (2011). Hydrothermal processing of hydrogen titanate/anatase-titania nanotubes and their application as strong dye-adsorbents. *J. Nanosci. Nanotechnol.* 11 1175–1187. 10.1166/jnn.2011.3048 21456156

[B110] Hasan J. Crawford R. J. Ivanova E. P. (2013). Antibacterial surfaces: the quest for a new generation of biomaterials. *Trends Biotechnol.* 31 295–304. 10.1016/j.tibtech.2013.01.017 23434154

[B111] He B. Yang Y. Yuen M. F. Chen X. F. Lee C. S. Zhang W. J. (2013). Vertical nanostructure arrays by plasma etching for applications in biology, energy, and electronics. *Nano Today* 8 265–289. 10.1016/j.nantod.2013.04.008

[B112] He L.-J. Hao J.-C. Dai L. Zeng R.-C. Li S.-Q. (2020). Layer-by-layer assembly of gentamicin-based antibacterial multilayers on Ti alloy. *Mater. Lett.* 261:127001 10.1016/j.matlet.2019.127001

[B113] He X. Zhang G. Wang X. Hang R. Huang X. Qin L. (2017). Biocompatibility, corrosion resistance and antibacterial activity of TiO2/CuO coating on titanium. *Ceram. Int.* 43 16185–16195. 10.1016/j.ceramint.2017.08.196

[B114] Heidarpour A. Aghamohammadi H. Ghasemi S. (2020). Structural and morphological characterization of the layered carbide-derived-carbon nanostructures obtained by HF etching of Ti2AlC. *Synth. Met.* 267:116478 10.1016/j.synthmet.2020.116478

[B115] Henam S. D. Ahmad F. Shah M. A. Parveen S. Wani A. H. (2019). Microwave synthesis of nanoparticles and their antifungal activities. *Spectrochim. Acta Part A Mol. Biomol. Spectrosc.* 213 337–341. 10.1016/j.saa.2019.01.071 30711904

[B116] Heo K. C. Gwag J. S. (2014). Shape-modification of patterned nanoparticles by an ion beam treatment. *Sci. Rep.* 5:8523. 10.1038/srep08523 25687920 PMC4330533

[B117] Hickok N. J. Shapiro I. M. (2012). Immobilized antibiotics to prevent orthopaedic implant infections. *Adv. Drug Deliv. Rev.* 64 1165–1176. 10.1016/j.addr.2012.03.015 22512927 PMC3413739

[B118] Hidalgo-Robatto B. M. López-Álvarez M. Azevedo A. S. Dorado J. Serra J. Azevedo N. F. (2018). Pulsed laser deposition of copper and zinc doped hydroxyapatite coatings for biomedical applications. *Surf. Coatings Technol.* 333 168–177. 10.1016/j.surfcoat.2017.11.006

[B119] Hirt L. Reiser A. Spolenak R. Zambelli T. (2017). Additive manufacturing of metal structures at the micrometer scale. *Adv. Mater.* 29:1604211. 10.1002/adma.201604211 28052421

[B120] Holleyman R. J. Deehan D. J. Walker L. Charlett A. Samuel J. Shirley M. D. F. (2019). Staphylococcal resistance profiles in deep infection following primary hip and knee arthroplasty: a study using the NJR dataset. *Arch. Orthop. Trauma Surg.* 139 1209–1215. 10.1007/s00402-019-03155-1 30877427 PMC6687688

[B121] Horkavcová D. Novák P. Èerný M. Jablonská E. Lipov J. (2017). Titania sol-gel coatings containing silver on newly developed TiSi alloys and their antibacterial effect. *Mater. Sci. Eng. C* 76 25–30. 10.1016/j.msec.2017.02.137 28482525

[B122] Hoyer P. (1996). Formation of a titanium dioxide nanotube array. *Langmuir* 12 1411–1413. 10.1021/la9507803

[B123] Huang J. Cheng Y. Wu Y. Shi X. Du Y. Deng H. (2019). Chitosan/tannic acid bilayers layer-by-layer deposited cellulose nanofibrous mats for antibacterial application. *Int. J. Biol. Macromol.* 139 191–198. 10.1016/j.ijbiomac.2019.07.185 31374279

[B124] Huang Y. Dan N. Dan W. Zhao W. Bai Z. Chen Y. (2019). Facile fabrication of gelatin and polycaprolactone based bilayered membranes via spin coating method with antibacterial and cyto-compatible properties. *Int. J. Biol. Macromol.* 124 699–707. 10.1016/j.ijbiomac.2018.11.262 30502434

[B125] Huang Y. He J. Gan L. Liu X. Wu Y. Wu F. (2014). Osteoconductivity and osteoinductivity of porous hydroxyapatite coatings deposited by liquid precursor plasma spraying: *in vivo* biological response study. *Biomed. Mater.* 9:065007 10.1088/1748-6041/9/6/065007 25384201

[B126] Huang Y. Xu Z. Zhang X. Chang X. Zhang X. Li Y. (2017). Nanotube-formed Ti substrates coated with silicate/silver co-doped hydroxyapatite as prospective materials for bone implants. *J. Alloys Compd.* 697 182–199. 10.1016/j.jallcom.2016.12.139

[B127] Hübsch C. Dellinger P. Maier H. J. Stemme F. Bruns M. Stiesch M. (2015). Protection of yttria-stabilized zirconia for dental applications by oxidic PVD coating. *Acta Biomater.* 11 488–493. 10.1016/j.actbio.2014.09.042 25278443

[B128] Huh A. J. Kwon Y. J. (2011). “Nanoantibiotics”: a new paradigm for treating infectious diseases using nanomaterials in the antibiotics resistant era. *J. Control. Release* 156 128–145. 10.1016/j.jconrel.2011.07.002 21763369

[B129] Huth M. Porrati F. Dobrovolskiy O. V. (2018). Focused electron beam induced deposition meets materials science. *Microelectron. Eng.* 185-186 9–28. 10.1016/j.mee.2017.10.012

[B130] Irshad M. A. Nawaz R. Zia ur Rehman M. Imran M. Ahmad J. Ahmad S. (2020). Synthesis and characterization of titanium dioxide nanoparticles by chemical and green methods and their antifungal activities against wheat rust. *Chemosphere* 258:127352. 10.1016/j.chemosphere.2020.127352 32554013

[B131] Ito T. Kunimatsu M. Kaneko S. Hirabayashi Y. Soga M. Agawa Y. (2012). High performance of hydrogen peroxide detection using Pt nanoparticles-dispersed carbon electrode prepared by pulsed arc plasma deposition. *Talanta* 99 865–870. 10.1016/j.talanta.2012.07.048 22967635

[B132] Ivanova E. P. Hasan J. Webb H. K. Gervinskas G. Juodkazis S. Truong V. K. (2013). Bactericidal activity of black silicon. *Nat. Commun.* 4:2838. 10.1038/ncomms3838 24281410 PMC3868328

[B133] Ivanova E. P. Hasan J. Webb H. K. Truong V. K. Watson G. S. Watson J. A. (2012). Natural bactericidal surfaces: mechanical rupture of *pseudomonas aeruginosa* cells by cicada wings. *Small* 8 2489–2494. 10.1002/smll.201200528 22674670

[B134] Jaggessar A. Mathew A. Wang H. Tesfamichael T. Yan C. Yarlagadda P. K. D. V. (2018). Mechanical, bactericidal and osteogenic behaviours of hydrothermally synthesised TiO2 nanowire arrays. *J. Mech. Behav. Biomed. Mater.* 80 311–319. 10.1016/j.jmbbm.2018.02.011 29459290

[B135] Jaggessar A. Yarlagadda P. K. D. V. (2020). Modelling the growth of hydrothermally synthesised bactericidal nanostructures, as a function of processing conditions. *Mater. Sci. Eng. C* 108:110434. 10.1016/j.msec.2019.110434 31924013

[B136] Jang J. Son M. Chung S. Kim K. Cho C. Lee B. H. (2015). Low-temperature-grown continuous graphene films from benzene by chemical vapor deposition at ambient pressure. *Sci. Rep.* 5:17955. 10.1038/srep17955 26658923 PMC4674705

[B137] Jawed S. F. Rabadia C. D. Liu Y. J. Wang L. Q. Li Y. H. Zhang X. H. (2019). Mechanical characterization and deformation behavior of β-stabilized Ti-Nb-Sn-Cr alloys. *J. Alloys Compd.* 792 684–693. 10.1016/j.jallcom.2019.04.079

[B138] Jin N. Yang Y. Luo X. Xia Z. (2013). Development of CVD Ti-containing films. *Prog. Mater. Sci.* 58 1490–1533. 10.1016/j.pmatsci.2013.07.001

[B139] Jones J. G. Voevodin A. A. (2004). Magnetron sputter pulsed laser deposition: technique and process control developments. *Surf. Coatings Technol.* 184 1–5. 10.1016/j.surfcoat.2003.10.016

[B140] Jun Y. Park J. H. Kang M. G. (2012). The preparation of highly ordered TiO2 nanotube arrays by an anodization method and their applications. *Chem. Commun.* 48 6456–6471. 10.1039/C2CC30733B 22634750

[B141] Jung J. H. Kobayashi H. van Bommel K. J. C. Shinkai S. Shimizu T. (2002). Creation of novel helical ribbon and double-layered nanotube TiO2 structures using an organogel template. *Chem. Mater.* 14 1445–1447. 10.1021/cm011625e

[B142] Karbowniczek J. Cordero-Arias L. Virtanen S. Misra S. K. Valsami-Jones E. Tuchscherr L. (2017). Electrophoretic deposition of organic/inorganic composite coatings containing ZnO nanoparticles exhibiting antibacterial properties. *Mater. Sci. Eng. C* 77 780–789. 10.1016/j.msec.2017.03.180 28532093

[B143] Kaur M. Singh K. (2019). Review on titanium and titanium based alloys as biomaterials for orthopaedic applications. *Mater. Sci. Eng. C* 102 844–862. 10.1016/j.msec.2019.04.064 31147056

[B144] Kaviyarasu K. Maria Magdalane C. Kanimozhi K. Kennedy J. Siddhardha B. Subba Reddy E. (2017). Elucidation of photocatalysis, photoluminescence and antibacterial studies of ZnO thin films by spin coating method. *J. Photochem. Photobiol. B Biol.* 173 466–475. 10.1016/j.jphotobiol.2017.06.026 28668515

[B145] Kelleher S. M. Habimana O. Lawler J. O’reilly B. Daniels S. Casey E. (2016). Cicada wing surface topography: an investigation into the bactericidal properties of nanostructural features. *ACS Appl. Mater. Interfaces* 8 14966–14974. 10.1021/acsami.5b08309 26551558

[B146] Khalandi B. Asadi N. Milani M. Davaran S. Abadi A. J. N. Abasi E. (2017). A review on potential role of silver nanoparticles and possible mechanisms of their actions on bacteria. *Drug Res.* 67 70–76. 10.1055/s-0042-113383 27824432

[B147] Khan F. A. (2020). “Nanomaterials: types, classifications, and sources,” in *Applications of Nanomaterials in Human Health*, ed. Khan F. (Singapore: Springer), 1–13. 10.1007/978-981-15-4802-4_1

[B148] Kheiri S. Liu X. Thompson M. (2019). Nanoparticles at biointerfaces: antibacterial activity and nanotoxicology. *Colloids Surfaces B Biointerfaces* 184:110550. 10.1016/j.colsurfb.2019.110550 31606698

[B149] Khoshnood N. Zamanian A. Massoudi A. (2017). Mussel-inspired surface modification of titania nanotubes as a novel drug delivery system. *Mater. Sci. Eng. C* 77 748–754. 10.1016/j.msec.2017.03.293 28532088

[B150] Khudhair D. Bhatti A. Li Y. Hamedani H. A. Garmestani H. Hodgson P. (2016). Anodization parameters influencing the morphology and electrical properties of TiO2 nanotubes for living cell interfacing and investigations. *Mater. Sci. Eng. C* 59 1125–1142. 10.1016/j.msec.2015.10.042 26652471

[B151] Kim H.-W. Koh Y.-H. Li L.-H. Lee S. Kim H.-E. (2004). Hydroxyapatite coating on titanium substrate with titania buffer layer processed by sol-gel method. *Biomaterials* 25 2533–2538. 10.1016/j.biomaterials.2003.09.041 14751738

[B152] Kim S. Park C. Cheon K.-H. Jung H.-D. Song J. Kim H.-E. (2018). Antibacterial and bioactive properties of stabilized silver on titanium with a nanostructured surface for dental applications. *Appl. Surf. Sci.* 451 232–240. 10.1016/j.apsusc.2018.04.270

[B153] Kim S. B. Jo J. H. Lee S. M. Kim H. E. Shin K. H. Koh Y. H. (2013). Use of a poly(ether imide) coating to improve corrosion resistance and biocompatibility of magnesium (Mg) implant for orthopedic applications. *J. Biomed. Mater. Res. art A* 101A 1708–1715. 10.1002/jbm.a.34474 23184807

[B154] Klein S. Nurjadi D. Eigenbrod T. Bode K. A. (2016). Evaluation of antibiotic resistance to orally administrable antibiotics in staphylococcal bone and joint infections in one of the largest university hospitals in Germany: Is there a role for fusidic acid? *Int. J. Antimicrob. Agents* 47 155–157. 10.1016/j.ijantimicag.2015.12.002 26774158

[B155] Kose N. Ayse Kose A. (2015). “Application of Nanomaterials in Prevention of Bone and Joint Infections,” in *Nanotechnology in Diagnosis, Treatment and Prophylaxis of Infectious Diseases*, eds M. Rai andK. Kon (Cambridge, MA: Academic Press), 107–117. 10.1016/B978-0-12-801317-5.00007-4

[B156] Kranthi Kiran A. S. Kizhakeyil A. Ramalingam R. Verma N. K. Lakshminarayanan R. Kumar T. S. S. (2019). Drug loaded electrospun polymer/ceramic composite nanofibrous coatings on titanium for implant related infections. *Ceram. Int.* 45 18710–18720. 10.1016/j.ceramint.2019.06.097

[B157] Krysina O. V. Prokopenko N. A. Ivanov Y. F. Tolkachev O. S. Shugurov V. V. Petrikova E. A. (2020). Multi-layered gradient (Zr,Nb)N coatings deposited by the vacuum-arc method. *Surf. Coatings Technol.* 393 125759 10.1016/j.surfcoat.2020.125759

[B158] Kumar A. Misra R. D. K. (2018). *3D-Printed Titanium Alloys for Orthopedic Applications.* (Amsterdam: Elsevier Inc). 10.1016/B978-0-12-812456-7.00012-3

[B159] Kumar S. Kumar P. Shan H. S. (2009). Characterization of the refractory coating material used in vacuum assisted evaporative pattern casting process. *J. Mater. Process. Technol.* 209 2699–2706. 10.1016/j.jmatprotec.2008.06.010

[B160] Kusano Y. Christou C. Barber Z. H. Evetts J. E. Hutchings I. M. (1999). Deposition of carbon nitride films by ionized magnetron sputtering. *Thin Solid Films* 355 117–121. 10.1016/S0040-6090(99)00510-6

[B161] Lai Y. Dong L. Zhou H. Yan B. Chen Y. Cai Y. (2020). Coexposed nanoparticulate Ag alleviates the acute toxicity induced by ionic Ag+ *in vivo*. *Sci. Total Environ.* 723:138050 10.1016/j.scitotenv.2020.138050 32217391

[B162] Laurent S. Bridot J. L. Elst L. Vander Muller R. N. (2010). Magnetic iron oxide nanoparticles for biomedical applications. *Future Med. Chem.* 2 427–449. 10.4155/fmc.09.164 21426176

[B163] Le Ouay B. Stellacci F. (2015). Antibacterial activity of silver nanoparticles: a surface science insight. *Nano Today* 10 339–354. 10.1016/j.nantod.2015.04.002

[B164] Lee J.-H. Leu I.-C. Hsu M.-C. Chung Y.-W. Hon M.-H. (2005). Fabrication of Aligned TiO2 one-dimensional nanostructured arrays using a one-step templating solution approach. *J. Phys. Chem. B* 109 13056–13059. 10.1021/jp052203l 16852622

[B165] Lee T. Mathew E. Rajaraman S. Manivasagam G. Singh A. K. Lee C. S. (2015). Tribological and corrosion behaviors of warm–and hot–rolled Ti–13Nb–13zr alloys in simulated body fluid conditions. *Int. J. Nanomed.* 10 207–212. 10.2147/IJN.S79996 26491322 PMC4599616

[B166] Lei T. Zhang W. Qian H. Lim P. N. Thian E. S. Lei P. (2020). Silicon-incorporated nanohydroxyapatite-reinforced poly(ε-caprolactone) film to enhance osteogenesis for bone tissue engineering applications. *Colloids Surfaces B Biointerfaces* 187:110714. 10.1016/j.colsurfb.2019.110714 31870518

[B167] Lelis M. Tuckute S. Varnagiris S. Urbonavicius M. Laukaitis G. Bockute K. (2019). Tailoring of TiO2 film microstructure by pulsed-DC and RF magnetron co-sputtering. *Surf. Coatings Technol.* 377:124906 10.1016/j.surfcoat.2019.124906

[B168] Li B. Webster T. J. (2018). Bacteria antibiotic resistance: new clant-associated orthopedic infehallenges and opportunities for impctions. *J. Orthop. Res.* 36 22–32. 10.1002/jor.23656 28722231 PMC5775060

[B169] Li D. Cheng Y. Jiang G. Yi Y. Shi X. (2019). Egg source natural proteins LBL modified cellulose nanofibrous mats and their cellular compatibility. *Carbohydr. Polym.* 213 329–337. 10.1016/j.carbpol.2019.02.096 30879676

[B170] Li D. Lv P. Fan L. Huang Y. Yang F. Mei X. (2017a). The immobilization of antibiotic-loaded polymeric coatings on osteoarticular Ti implants for the prevention of bone infections. *Biomater. Sci.* 5 2337–2346. 10.1039/c7bm00693d 29034380

[B171] Li H. Feng X. Peng Y. Zeng R. (2020). Durable lubricant-infused coating on a magnesium alloy substrate with anti-biofouling and anti-corrosion properties and excellent thermally assisted healing ability. *Nanoscale* 12 7700–7711. 10.1039/c9nr10699e 32211633

[B172] Li H. F. Nie F. L. Zheng Y. F. Cheng Y. Wei S. C. Valiev R. Z. (2019). Nanocrystalline Ti49.2*Ni*50.8 shape memory alloy as orthopaedic implant material with better performance. *J. Mater. Sci. Technol.* 35 2156–2162. 10.1016/j.jmst.2019.04.026

[B173] Li J. Li Q. Ma X. Tian B. Li T. Yu J. (2016). Biosynthesis of gold nanoparticles by the extreme bacterium Deinococcus radiodurans and an evaluation of their antibacterial properties. *Int. J. Nanomed.* 11 5931–5944. 10.2147/IJN.S119618 27877039 PMC5108609

[B174] Li J. Tan L. Liu X. Cui Z. Yang X. Yeung K. W. K. (2017b). Balancing Bacteria-Osteoblast Competition through Selective Physical Puncture and Biofunctionalization of ZnO/Polydopamine/Arginine-Glycine-Aspartic Acid-Cysteine Nanorods. *ACS Nano* 11 11250–11263. 10.1021/acsnano.7b05620 29049874

[B175] Li J. Wang Y. Yao Y. Wang Y. Wang L. (2017c). Structure and tribological properties of TiSiCN coating on Ti6Al4V by arc ion plating. *Thin Solid Films* 644 115–119. 10.1016/j.tsf.2017.09.053

[B176] Li W. W. Wang H. Y. Zhang Y. Q. (2017d). A novel chitosan hydrogel membrane by an improved electrophoretic deposition and its characteristics *in vitro* and *in vivo*. *Mater. Sci. Eng. C* 74 287–297. 10.1016/j.msec.2016.12.005 28254297

[B177] Li Y. Zimmerman A. R. He F. Chen J. Han L. Chen H. (2020). Solvent-free synthesis of magnetic biochar and activated carbon through ball-mill extrusion with Fe3O4 nanoparticles for enhancing adsorption of methylene blue. *Sci. Total Environ.* 722:137972. 10.1016/j.scitotenv.2020.137972 32208286

[B178] Liang R. Xu Y. Zhao M. Han G. Li J. Wu W. (2020). Properties of silver contained coatings on CoCr alloys prepared by vacuum plasma spraying. *Mater. Sci. Eng. C* 106:110156. 10.1016/j.msec.2019.110156 31753375

[B179] Liang S. X. Feng X. J. Yin L. X. Liu X. Y. Ma M. Z. Liu R. P. (2016). Development of a new β Ti alloy with low modulus and favorable plasticity for implant material. *Mater. Sci. Eng. C* 61 338–343. 10.1016/j.msec.2015.12.076 26838858

[B180] Liao C. Wuethrich A. Trau M. (2020). A material odyssey for 3D nano/microstructures: two photon polymerization based nanolithography in bioapplications. *Appl. Mater. Today* 19:100635 10.1016/j.apmt.2020.100635

[B181] Limoli D. H. Jones C. J. Wozniak D. J. (2015). Bacterial extracellular polysaccharides in biofilm formation and function. *Microb. Biofilms* 223–247. 10.1128/9781555817466.ch11 PMC4657554 26185074

[B182] Lin J. Chen H. Fei T. Zhang J. (2013). Highly transparent superhydrophobic organic-inorganic nanocoating from the aggregation of silica nanoparticles. *Colloids Surfaces A Physicochem. Eng. Asp.* 421 51–62. 10.1016/j.colsurfa.2012.12.049

[B183] Linklater D. P. De Volder M. Baulin V. A. Werner M. Jessl S. Golozar M. (2018). High aspect ratio nanostructures kill bacteria via storage and release of mechanical energy. *ACS Nano* 12 6657–6667. 10.1021/acsnano.8b01665 29851466

[B184] Linklater D. P. Juodkazis S. Crawford R. J. Ivanova E. P. (2019). Mechanical inactivation of *Staphylococcus aureus* and *Pseudomonas aeruginosa* by titanium substrata with hierarchical surface structures. *Materialia* 5:100197 10.1016/j.mtla.2018.100197

[B185] Linklater D. P. Juodkazis S. Rubanov S. Ivanova E. P. (2017). Comment on “bactericidal Effects of Natural Nanotopography of Dragonfly Wing on *Escherichia coli*.”. *ACS Appl. Mater. Interfaces* 9 29387–29393. 10.1021/acsami.7b05707 28799744

[B186] Liu D. He C. Liu Z. Xu W. (2017). Gentamicin coating of nanotubular anodized titanium implant reduces implant-related osteomyelitis and enhances bone biocompatibility in rabbits. *Int. J. Nanomed.* 12 5461–5471. 10.2147/IJN.S137137 28814863 PMC5546782

[B187] Liu J. Hurt R. H. (2010). Ion release kinetics and particle persistence in aqueous nano-silver colloids. *Environ. Sci. Technol.* 44 2169–2175. 10.1021/es9035557 20175529

[B188] Liu N. Chen X. Zhang J. Schwank J. W. (2014). A review on TiO2-based nanotubes synthesized via hydrothermal method: formation mechanism, structure modification, and photocatalytic applications. *Catal. Today* 225 34–51. 10.1016/j.cattod.2013.10.090

[B189] Liu R. Ma Z. Kolawole S. Zeng L. Zhao Y. Ren L. (2019). *In vitro* study on cytocompatibility and osteogenesis ability of Ti–Cu alloy. *J. Mater. Sci. Mater. Med.* 30:75. 10.1007/s10856-019-6277-z 31218519

[B190] Liu W. Cheng M. Wahafu T. Zhao Y. Qin H. Wang J. (2015a). The *in vitro* and *in vivo* performance of a strontium-containing coating on the low-modulus Ti35Nb2Ta3Zr alloy formed by micro-arc oxidation. *J. Mater. Sci. Mater. Med.* 26:203. 10.1007/s10856-015-5533-0 26152510

[B191] Liu W. Su P. Gonzales A. Chen S. Wang N. Wang J. (2015b). Optimizing stem cell functions and antibacterial properties of TiO2 nanotubes incorporated with ZnO nanoparticles: experiments and modeling. *Int. J. Nanomed.* 10 1997–2019. 10.2147/IJN.S74418 25792833 PMC4364596

[B192] Liu X. Man H. C. (2017). Laser fabrication of Ag-HA nanocomposites on Ti6Al4V implant for enhancing bioactivity and antibacterial capability. *Mater. Sci. Eng. C* 70 1–8. 10.1016/j.msec.2016.08.059 27770868

[B193] Liu Y. Hang R. Zhao Y. Bai L. Sun Y. Yao X. (2018). The effects of annealing temperature on corrosion behavior, Ni2+ release, cytocompatibility, and antibacterial ability of Ni-Ti-O nanopores on NiTi alloy. *Surf. Coatings Technol.* 352 175–181. 10.1016/j.surfcoat.2018.08.016

[B194] Lü X. Bao X. Huang Y. Qu Y. Lu H. Lu Z. (2009). Mechanisms of cytotoxicity of nickel ions based on gene expression profiles. *Biomaterials* 30 141–148. 10.1016/j.biomaterials.2008.09.011 18922574

[B195] Luo X. Yao S. Zhang H. Cai M. Liu W. Pan R. (2020). Biocompatible nano-ripples structured surfaces induced by femtosecond laser to rebel bacterial colonization and biofilm formation. *Opt. Laser Technol.* 124:105973 10.1016/j.optlastec.2019.105973

[B196] Luo Z. M. Wang J. W. Tan J. B. Zhang Z. M. Lu T. B. (2018). Self-Template Synthesis of Co-Se-S-O Hierarchical Nanotubes as Efficient Electrocatalysts for Oxygen Evolution under Alkaline and Neutral Conditions. *ACS Appl. Mater. Interfaces* 10 8231–8237. 10.1021/acsami.8b00986 29433305

[B197] Lv P. Zhu L. Yu Y. Wang W. Liu G. Lu H. (2020). Effect of NaOH concentration on antibacterial activities of Cu nanoparticles and the antibacterial mechanism. *Mater. Sci. Eng. C* 110:110669 10.1016/j.msec.2020.110669 32204097

[B198] Lv Y. Lu X. Wu Y. Yu Y. Fu S. Yang L. (2019). Microstructure, bio-corrosion and biological property of Ag-incorporated TiO2 coatings: influence of Ag2O contents. *Ceram. Int.* 45 22357–22367. 10.1016/j.ceramint.2019.07.265

[B199] Ma B. (2007). *Porous Structured Titania by Chemical Methods.* Ph.D. thesis, Nanyang Technological University, Singapore 10.32657/10356/5088

[B200] Macak J. M. Tsuchiya H. Taveira L. Ghicov A. Schmuki P. (2005). Self-organized nanotubular oxide layers on Ti-6Al-7Nb and Ti-6Al-4V formed by anodization in NH4F solutions. *J. Biomed. Mater. Res. Part A* 75 928–933. 10.1002/jbm.a.30501 16138327

[B201] Mageswari A. Srinivasan R. Subramanian P. Ramesh N. Gothandam K. M. (2016). Nanomaterials: classification, biological synthesis and characterization. *Nanosci. Food Agric.* 3 31–71. 10.1007/978-3-319-48009-1_2

[B202] Mahade S. Narayan K. Govindarajan S. Björklund S. Curry N. Joshi S. (2019). Exploiting suspension plasma spraying to deposit wear-resistant carbide coatings. *Materials* 12:2344. 10.3390/ma12152344 31344804 PMC6696411

[B203] Mahlooji E. Atapour M. Labbaf S. (2019). Electrophoretic deposition of Bioactive glass – Chitosan nanocomposite coatings on Ti-6Al-4V for orthopedic applications. *Carbohydr. Polym.* 226:115299. 10.1016/j.carbpol.2019.115299 31582073

[B204] Maimaiti B. Zhang N. Yan L. Luo J. Xie C. Wang Y. (2020). Stable ZnO-doped hydroxyapatite nanocoating for anti-infection and osteogenic on titanium. *Colloids Surfaces B Biointerfaces* 186:110731. 10.1016/j.colsurfb.2019.110731 31855685

[B205] Makówka M. Pawlak W. Konarski P. Wendler B. Szymanowski H. (2019). Modification of magnetron sputter deposition of nc-WC/a-C(:H) coatings with an additional RF discharge. *Diam. Relat. Mater.* 98:107509 10.1016/j.diamond.2019.107509

[B206] Malhotra R. Han Y. M. Morin J. L. P. Luong-Van E. K. Chew R. J. J. Castro Neto A. H. (2020). Inhibiting corrosion of biomedical-grade Ti-6Al-4V alloys with graphene nanocoating. *J. Dent. Res.* 99 285–292. 10.1177/0022034519897003 31905311

[B207] Manawi Y. M. Samara A. Al-Ansari T. Atieh M. A. (2018). A Review of Carbon Nanomaterials’ Synthesis via the Chemical Vapor Deposition (CVD) Method. *Materials* 11:822. 10.3390/ma11050822 29772760 PMC5978199

[B208] Marambio-Jones C. Hoek E. M. V. (2010). A review of the antibacterial effects of silver nanomaterials and potential implications for human health and the environment. *J. Nanoparticle Res.* 12 1531–1551. 10.1007/s11051-010-9900-y

[B209] Masuda H. Fukuda K. (1995). Ordered metal nanohole arrays made by a two-step replication of honeycomb structures of anodic alumina. *Science* 268 1466–1468. 10.1126/science.268.5216.1466 17843666

[B210] Maury F. Senocq F. (2003). Iridium coatings grown by metal-organic chemical vapor deposition hot-wall CVD reactor. *Surf. Coatings Technol.* 163–164 208–213. 10.1016/S0257-8972(02)00485-1

[B211] Mazare A. Anghel A. Carmen S.-B. Totea G. Ionita D. (2018). Silver doped diamond-like carbon antibacterial and corrosion resistance coatings on titanium. *Thin Solid Films* 657 16–23 . 10.1016/j.tsf.2018.04.036

[B212] Meng L. Y. Wang B. Ma M. G. Lin K. L. (2016). The progress of microwave-assisted hydrothermal method in the synthesis of functional nanomaterials. *Mater. Today Chem.* 1 63–83. 10.1016/j.mtchem.2016.11.003

[B213] Mi B. Xiong W. Xu N. Guan H. Fang Z. Liao H. (2017). Strontium-loaded titania nanotube arrays repress osteoclast differentiation through multiple signalling pathways: *in vitro* and *in vivo* studies. *Sci. Rep.* 7:2328. 10.1038/s41598-017-02491-9 28539667 PMC5443803

[B214] Mi G. Shi D. Wang M. Webster T. J. (2018). Reducing bacterial infections and biofilm formation using nanoparticles and nanostructured antibacterial surfaces. *Adv. Healthc. Mater.* 7 1–23. 10.1002/adhm.201800103 29790304

[B215] Miao H. Hu X. Fan J. Li C. Sun Q. Hao Y. (2015). Hydrothermal synthesis of TiO 2 nanostructure films and their photoelectrochemical properties. *Appl. Surf. Sci.* 358, 418–424. 10.1016/j.apsusc.2015.08.212

[B216] Minagar S. Berndt C. C. Gengenbach T. Wen C. (2014). Fabrication and characterization of TiO2-ZrO2- ZrTiO4 nanotubes on TiZr alloy manufactured via anodization. *J. Mater. Chem. B* 2 71–83. 10.1039/c3tb21204a 32261300

[B217] Modaresifar K. Azizian S. Ganjian M. Fratila-Apachitei L. E. Zadpoor A. A. (2019). Bactericidal effects of nanopatterns: a systematic review. *Acta Biomater.* 83 29–36. 10.1016/j.actbio.2018.09.059 30273746

[B218] Mohan L. Dennis C. Padmapriya N. Anandan C. Rajendran N. (2020). Effect of electrolyte temperature and anodization time on formation of TiO2 nanotubes for biomedical applications. *Mater. Today Commun.* 23:101103 10.1016/j.mtcomm.2020.101103

[B219] Mohan Raj R. Priya P. Raj V. (2018). Gentamicin-loaded ceramic-biopolymer dual layer coatings on the Ti with improved bioactive and corrosion resistance properties for orthopedic applications. *J. Mech. Behav. Biomed. Mater.* 82 299–309. 10.1016/j.jmbbm.2017.12.033 29649658

[B220] Molleman B. Hiemstra T. (2015). Surface structure of silver nanoparticles as a model for understanding the oxidative dissolution of silver ions. *Langmuir* 31 13361–13372. 10.1021/acs.langmuir.5b03686 26595806

[B221] Montanaro L. Speziale P. Campoccia D. Ravaioli S. Pietrocola G. (2011). Scenery of Staphylococcus implant infections in orthopedics. *Future Microbiol.* 6 1329–1349. 10.2217/fmb.11.117 22082292

[B222] Moreira A. J. Campos L. O. Maldi C. P. Dias J. A. Paris E. C. Giraldi T. R. (2020). Photocatalytic degradation of Prozac^®^ mediated by TiO2 nanoparticles obtained via three synthesis methods: sonochemical, microwave hydrothermal, and polymeric precursor. *Environ. Sci. Pollut. Res.* 27 27032–27047. 10.1007/s11356-020-08798-x 32388756

[B223] Morones-Ramirez J. Elechiguerra J. Camacho A. Holt K. Kouri J. Tapia J. (2005). The bactericidal effect of silver nanoparticles. *Nanotechnology* 16 2346–2353. 10.1088/0957-4484/16/10/059 20818017

[B224] Morris J. E. (2018). “Nanoparticle properties,” in *Nanopackaging*, 2nd Edn, ed. Morris J. E. (Cham: Springer), 201–217. 10.1007/978-3-319-90362-0_6

[B225] Mostaghimi J. Chandra S. (2007). Heat transfer in plasma spray coating processes. *Adv. Heat Transf.* 40 143–204. 10.1016/S0065-2717(07)40002-8

[B226] Mou P. Peng H. Zhou L. Li L. Li H. Huang Q. (2019). A novel composite scaffold of Cu-doped nano calcium-deficient hydroxyapatite/multi-(amino acid) copolymer for bone tissue regeneration. *Int. J. Nanomed.* 14 3331–3343. 10.2147/IJN.S195316 31123401 PMC6511241

[B227] Multone X. Luo Y. Hoffmann P. (2008). Er-doped Al2O3 thin films deposited by high-vacuum chemical vapor deposition (HV-CVD). *Mater. Sci. Eng. B Solid State Mater. Adv. Technol.* 146 35–40. 10.1016/j.mseb.2007.07.086

[B228] Nakahira A. Kubo T. Numako C. (2010). Formation mechanism of TiO2-derived titanate nanotubes prepared by the hydrothermal process. *Inorg. Chem.* 49 5845–5852. 10.1021/ic9025816 20527822

[B229] Newland B. Taplan C. Pette D. Friedrichs J. Steinhart M. Wang W. (2018). Soft and flexible poly(ethylene glycol) nanotubes for local drug delivery. *Nanoscale* 10 8413–8421. 10.1039/C8NR00603B 29714385 PMC5944428

[B230] Nguyen A. N. Solard J. Nong H. T. T. Osman C. Ben, Gomez A. (2020). Spin coating and micro-patterning optimization of composite thin films based on PVDF. *Materials* 13:1342. 10.3390/ma13061342 32187993 PMC7143455

[B231] Nguyen-Tri P. Tran H. N. Plamondon C. O. Tuduri L. Vo D. V. N. Nanda S. (2019). Recent progress in the preparation, properties and applications of superhydrophobic nano-based coatings and surfaces: a review. *Prog. Org. Coatings* 132 235–256. 10.1016/j.porgcoat.2019.03.042

[B232] Ni J. Frandsen C. J. Noh K. Johnston G. W. He G. Tang T. (2013). Fabrication of thin film TiO2 nanotube arrays on Co-28Cr-6Mo alloy by anodization. *Mater. Sci. Eng. C* 33 1460–1466. 10.1016/j.msec.2012.12.068 23827596 PMC4040976

[B233] Ning C. Jiajia J. Meng L. Hongfei Q. Xianglong W. Tingli L. (2019). Electrophoretic deposition of GHK-Cu loaded MSN-chitosan coatings with pH-responsive release of copper and its bioactivity. *Mater. Sci. Eng. C* 104:109746. 10.1016/j.msec.2019.109746 31500015

[B234] Nowlin K. Boseman A. Covell A. LaJeunesse D. (2014). Adhesion-dependent rupturing of Saccharomyces cerevisiae on biological antimicrobial nanostructured surfaces. *J. R. Soc. Interface* 12:20140999. 10.1098/rsif.2014.0999 25551144 PMC4277089

[B235] Ogunyemi S. O. Abdallah Y. Zhang M. Fouad H. Hong X. Ibrahim E. (2019). Green synthesis of zinc oxide nanoparticles using different plant extracts and their antibacterial activity against *Xanthomonas oryzae* pv. *oryzae*. *Artif. Cells Nanomed. Biotechnol.* 47 341–352. 10.1080/21691401.2018.1557671 30691311

[B236] Ohtsu N. Suginishi S. Hirano M. (2017). Antibacterial effect of nickel-titanium alloy owing to nickel ion release. *Appl. Surf. Sci.* 405 215–219. 10.1016/j.apsusc.2017.02.037

[B237] Ovsianikov A. Chichkov B. N. (2012). Three-dimensional microfabrication by two-photon polymerization technique. *Methods Mol. Biol.* 868 311–325. 10.1007/978-1-61779-764-4_19 22692619

[B238] Oytun F. Basarir F. (2019). Spin-assisted layer-by-layer assembled oppositely charged reduced graphene oxide films. *Mater. Lett.* 257:126756 10.1016/j.matlet.2019.126756

[B239] Pagedar A. Singh J. Batish V. K. (2010). Surface hydrophobicity, nutritional contents affect Staphylococcus aureus biofilms and temperature influences its survival in preformed biofilms. *J. Basic Microbiol.* 50 S98–S106. 10.1002/jobm.201000034 20586075

[B240] Pandey J. K. Swarnkar R. K. Soumya K. K. Dwivedi P. Singh M. K. Sundaram S. (2014). Silver nanoparticles synthesized by pulsed laser ablation: as a potent antibacterial agent for human enteropathogenic gram-positive and gram-negative bacterial strains. *Appl. Biochem. Biotechnol.* 174 1021–1031. 10.1007/s12010-014-0934-y 24801405

[B241] Paneerselvam E. Vasa N. J. Nakamura D. Palani I. A. Higashihata M. Ramachandra Rao M. S. (2020). Pulsed laser deposition of SiC thin films and influence of laser-assisted annealing. *Mater. Today Proc.* 10.1016/j.matpr.2020.01.535

[B242] Pang S. He Y. Zhong R. Guo Z. He P. Zhou C. (2019). Multifunctional ZnO/TiO 2 nanoarray composite coating with antibacterial activity, cytocompatibility and piezoelectricity. *Ceram. Int.* 45 12663–12671. 10.1016/j.ceramint.2019.03.076

[B243] Pareek V. Gupta R. Panwar J. (2018). Do physico-chemical properties of silver nanoparticles decide their interaction with biological media and bactericidal action? A review. *Mater. Sci. Eng. C* 90 739–749. 10.1016/j.msec.2018.04.093 29853145

[B244] Park H.-J. Kim J. Y. Kim J. Lee J.-H. Hahn J.-S. Gu M. B. (2009). Silver-ion-mediated reactive oxygen species generation affecting bactericidal activity. *Water Res.* 43 1027–1032. 10.1016/j.watres.2008.12.002 19073336

[B245] Patil D. Wasson M. K. Aravindan S. Vivekanandan P. Rao P. V. (2019). Antibacterial and cytocompatibility study of modified Ti6Al4V surfaces through thermal annealing. *Mater. Sci. Eng. C* 99 1007–1020. 10.1016/j.msec.2019.02.058 30889633

[B246] Perentes A. Bachmann A. Leutenegger M. Sandu C. (2004). Focused electron beam induced deposition of a periodic transparent nano-optic pattern. *Microelectron. Eng.* 73–74 412–416. 10.1016/j.mee.2004.02.079

[B247] Persat A. (2017). Bacterial mechanotransduction. *Curr. Opin. Microbiol.* 36 1–6. 10.1016/j.mib.2016.12.002 28068612

[B248] Pfang B. G. García-Cañete J. García-Lasheras J. Blanco A. Auñón Á. Parron-Cambero R. (2019). Orthopedic implant-associated infection by multidrug resistant *Enterobacteriaceae*. *J. Clin. Med.* 8:220. 10.3390/jcm8020220 30744054 PMC6406851

[B249] Pinna E. Le Gall S. Torralba E. Mula G. Cachet-Vivier C. Bastide S. (2020). Mesopore formation and silicon surface nanostructuration by metal-assisted chemical etching with silver nanoparticles. *Front. Chem.* 8:658. 10.3389/fchem.2020.00658 32850670 PMC7416550

[B250] Pishbin F. Mouriño V. Gilchrist J. B. McComb D. W. Kreppel S. Salih V. (2013). Single-step electrochemical deposition of antimicrobial orthopaedic coatings based on a bioactive glass/chitosan/nano-silver composite system. *Acta Biomater.* 9 7469–7479. 10.1016/j.actbio.2013.03.006 23511807

[B251] Pišlová M. Koláøová K. Vokatá B. Brož A. Ulbrich P. Bačáková L. (2020). A new way to prepare gold nanoparticles by sputtering – Sterilization, stability and other properties. *Mater. Sci. Eng. C* 115:111087. 10.1016/j.msec.2020.111087 32600693

[B252] Pruchova E. Kosova M. Fojt J. Jarolimova P. Jablonska E. Hybasek V. (2019). A two-phase gradual silver release mechanism from a nanostructured TiAlV surface as a possible antibacterial modification in implants. *Bioelectrochemistry* 127 26–34. 10.1016/j.bioelechem.2019.01.003 30654242

[B253] Qian H. Lei T. Lei P. E. Hu Y. (2020). Additively manufactured Tantalum implants for repairing bone defects: a systematic review. *Tissue Eng. Part B Rev.* 10.1089/ten.teb.2020.0134 [Epub ahead of print). 32799765

[B254] Quinteros M. A. Cano Aristizábal V. Dalmasso P. R. Paraje M. G. Páez P. L. (2016). Oxidative stress generation of silver nanoparticles in three bacterial genera and its relationship with the antimicrobial activity. *Toxicol. In Vitro* 36 216–223. 10.1016/j.tiv.2016.08.007 27530963

[B255] Rabadia C. D. Liu Y. J. Chen L. Y. Jawed S. F. Wang L. Q. Sun H. (2019a). Deformation and strength characteristics of Laves phases in titanium alloys. *Mater. Des.* 179:107891 10.1016/j.matdes.2019.107891

[B256] Rabadia C. D. Liu Y. J. Zhao C. H. Wang J. C. Jawed S. F. Wang L. Q. (2019b). Improved trade-off between strength and plasticity in titanium based metastable beta type Ti-Zr-Fe-Sn alloys. *Mater. Sci. Eng. A* 766:138340 10.1016/j.msea.2019.138340

[B257] Rafieerad A. R. Bushroa A. R. Nasiri-Tabrizi B. Baradaran S. Amiri A. Saber-Samandari S. (2019). Simultaneous enhanced antibacterial and osteoblast cytocompatibility performance of Ti6Al7Nb implant by nano-silver/graphene oxide decorated mixed oxide nanotube composite. *Surf. Coatings Technol.* 360 181–195. 10.1016/j.surfcoat.2018.12.119

[B258] Rahnamaee S. Y. Bagheri R. Vossoughi M. Ahmadi Seyedkhani S. Samadikuchaksaraei A. (2020). Bioinspired multifunctional TiO2 hierarchical micro/nanostructures with tunable improved bone cell growth and inhibited bacteria adhesion. *Ceram. Int.* 46 9669–9679. 10.1016/j.ceramint.2019.12.234

[B259] Rajesh S. Zhao Y. Fong H. Menkhaus T. J. (2016). Polyacrylonitrile nanofiber membranes modified with ionically crosslinked polyelectrolyte multilayers for the separation of ionic impurities. *Nanoscale* 8 18376–18389. 10.1039/c6nr06295d 27766338

[B260] Ramalingam B. Parandhaman T. Das S. K. (2016). Antibacterial Effects of Biosynthesized Silver Nanoparticles on Surface Ultrastructure and Nanomechanical Properties of Gram-Negative Bacteria viz. *Escherichia coli and Pseudomonas aeruginosa*. *ACS Appl. Mater. Interfaces* 8 4963–4976. 10.1021/acsami.6b00161 26829373

[B261] Ranjbar Z. Rastegar S. (2011). Nano mechanical properties of an automotive clear-coats containing nano silica particles with different surface chemistries. *Prog. Org. Coatings* 72 40–43. 10.1016/j.porgcoat.2010.11.001

[B262] Ranoszek-Soliwoda K. Tomaszewska E. Małek K. Celichowski G. Orlowski P. Krzyzowska M. (2019). The synthesis of monodisperse silver nanoparticles with plant extracts. *Colloids Surfaces B Biointerfaces* 177 19–24. 10.1016/j.colsurfb.2019.01.037 30690426

[B263] Ratova M. Klaysri R. Praserthdam P. Kelly P. J. (2017). Pulsed DC magnetron sputtering deposition of crystalline photocatalytic titania coatings at elevated process pressures. *Mater. Sci. Semicond. Process.* 71 188–196. 10.1016/j.mssp.2017.07.028

[B264] Rauf A. Ye J. Zhang S. Qi Y. Wang G. Che Y. (2019). Copper(ii)-based coordination polymer nanofibers as a highly effective antibacterial material with a synergistic mechanism. *Dalt. Trans.* 48 17810–17817. 10.1039/C9DT03649K 31773125

[B265] Ribbens S. Meynen V. Tendeloo G. Van, Ke X. Mertens M. (2008). Development of photocatalytic efficient Ti-based nanotubes and nanoribbons by conventional and microwave assisted synthesis strategies. *Microporous Mesoporous Mater.* 114 401–409. 10.1016/j.micromeso.2008.01.028

[B266] Riedesel C. Kaufmann N. Adolf A. Kaemmer N. Fritz H. (2019). “First demonstration of a 331-beam SEM,” in *Proceedings of the SPIE 10959, Metrology, Inspection, and Process Control for Microlithography XXXIII*, San Jose, CA 10.1117/12.2528795

[B267] Rizzello L. Pompa P. P. (2014). Nanosilver-based antibacterial drugs and devices: mechanisms, methodological drawbacks, and guidelines. *Chem. Soc. Rev.* 43 1501–1518. 10.1039/c3cs60218d 24292075

[B268] Rizzello L. Sorce B. Sabella S. Vecchio G. Galeone A. Brunetti V. (2011). Impact of nanoscale topography on genomics and proteomics of adherent bacteria. *ACS Nano* 5 1865–1876. 10.1021/nn102692m 21344880

[B269] Roman I. Trusca R. D. Soare M.-L. Fratila C. Krasicka-Cydzik E. Stan M. (2014). Titanium dioxide nanotube films: preparation, characterization and electrochemical biosensitivity towards alkaline phosphatase. *Mater. Sci. Eng. C* 37 374–382. 10.1016/j.msec.2014.01.036 24582263

[B270] Rosifini Alves Claro A. P. Konatu R. T. do Amaral Escada A. L. de Souza Nunes M. C. Maurer-Morelli C. V. Dias-Netipanyj M. F. (2018). Incorporation of silver nanoparticles on Ti7.5Mo alloy surface containing TiO2 nanotubes arrays for promoting antibacterial coating – *in vitro* and *in vivo* study. *Appl. Surf. Sci.* 455 780–788.

[B271] Sabirov I. Enikeev N. A. Murashkin M. Y. Valiev R. Z. (2015). *Nanostructures in Materials Subjected to Severe Plastic Deformation.* (Cham: Springer), 11–26. 10.1007/978-3-319-19599-5_2

[B272] Saleh T. A. (2020). Nanomaterials: classification, properties, and environmental toxicities. *Environ. Technol. Innov.* 20:101067 10.1016/j.eti.2020.101067

[B273] Salwiczek M. Qu Y. Gardiner J. Strugnell R. A. Lithgow T. McLean K. M. (2014). Emerging rules for effective antimicrobial coatings. *Trends Biotechnol.* 32 82–90. 10.1016/j.tibtech.2013.09.008 24176168

[B274] Sam Froes F. H. (2018). Titanium for medical and dental applications-An introduction. *Titan. Med. Dent. Appl.* 3–21. 10.1016/B978-0-12-812456-7.00001-9

[B275] Samanta A. Podder S. Kumarasamy M. Ghosh C. K. Lahiri D. Roy P. (2019). Au nanoparticle-decorated aragonite microdumbbells for enhanced antibacterial and anticancer activities. *Mater. Sci. Eng. C Mater. Biol. Appl.* 103:109734. 10.1016/j.msec.2019.05.019 31349529

[B276] Sano K. Kuttassery F. Shimada T. Ishida T. Takagi S. Ohtani B. (2020). Optically Transparent Colloidal Dispersion of TiO2 Nanoparticles Storable for longer than One year Prepared by Sol/Gel Progressive Hydrolysis/Condensation. *ACS Appl. Mater. Interfaces* 12 44743–44753. 10.1021/acsami.0c12951 32915534

[B277] Sarkari Khorrami M. Saito N. Miyashita Y. Kondo M. (2019). Texture variations and mechanical properties of aluminum during severe plastic deformation and friction stir processing with SiC nanoparticles. *Mater. Sci. Eng. A* 744 349–364. 10.1016/j.msea.2018.12.031

[B278] Sarraf M. Dabbagh A. Abdul Razak B. Mahmoodian R. Nasiri-Tabrizi B. Hosseini H. R. M. (2018a). Highly-ordered TiO2 nanotubes decorated with Ag2O nanoparticles for improved biofunctionality of Ti6Al4V. *Surf. Coatings Technol.* 349 1008–1017. 10.1016/j.surfcoat.2018.06.054

[B279] Sarraf M. Dabbagh A. Abdul Razak B. Nasiri-Tabrizi B. Hosseini H. R. M. Saber-Samandari S. (2018b). Silver oxide nanoparticles-decorated tantala nanotubes for enhanced antibacterial activity and osseointegration of Ti6Al4V. *Mater. Des.* 154 28–40. 10.1016/j.matdes.2018.05.025

[B280] Sarró M. I. Moreno D. A. Ranninger C. King E. Ruiz J. (2006). Influence of gas nitriding of Ti6Al4V alloy at high temperature on the adhesion of Staphylococcus aureus. *Surf. Coatings Technol.* 201 2807–2812. 10.1016/j.surfcoat.2006.05.023

[B281] Scott C. Olier C. Lamandé A. Choquet P. Chaleix D. (2003). Structural evolution of co-deposited Zn-Cr coatings produced by vacuum evaporation. *Thin Solid Films* 436 232–237. 10.1016/S0040-6090(03)00597-2

[B282] Semenova I. P. Polyakov A. V. Polyakova V. V. Grishina Y. F. Huang Y. Valiev R. Z. (2017). Mechanical behavior and impact toughness of the ultrafine-grained Grade 5 Ti alloy processed by ECAP. *Mater. Sci. Eng. A* 696 166–173. 10.1016/j.msea.2017.04.073

[B283] Seo D. S. Lee J. K. Kim H. (2001). Preparation of nanotube-shaped TiO2 powder. *J. Cryst. Growth* 229 428–432. 10.1016/S0022-0248(01)01196-4

[B284] Seong M. Lee D. G. (2017). Silver nanoparticles against *Salmonella enterica* Serotype typhimurium: role of inner membrane dysfunction. *Curr. Microbiol.* 74 661–670. 10.1007/s00284-017-1235-9 28321528

[B285] Sergi R. Bellucci D. Candidato R. T. Lusvarghi L. Bolelli G. Pawlowski L. (2018). Bioactive Zn-doped hydroxyapatite coatings and their antibacterial efficacy against *Escherichia coli* and Staphylococcus aureus. *Surf. Coatings Technol.* 352 84–91. 10.1016/j.surfcoat.2018.08.017

[B286] Shang F. Chen S. Liang J. Liu C. (2019). Preparation and Photocatalytic Properties of ZnO Deposited TiO(2) Nanotube Arrays by Anodization. *J. Nanosci. Nanotechnol.* 19 2070–2077. 10.1166/jnn.2019.15797 30486949

[B287] Shao S. Y. Chen J. X. Tang H. Y. Ming P. P. Yang J. Zhu W. Q. (2020). A titanium surface modified with zinc-containing nanowires: enhancing biocompatibility and antibacterial property *in vitro*. *Appl. Surf. Sci.* 515:146107 10.1016/j.apsusc.2020.146107

[B288] Shuitcev A. Gunderov D. V. Sun B. Li L. Valiev R. Z. Tong Y. X. (2020). Nanostructured Ti29.7Ni50.3Hf20 high temperature shape memory alloy processed by high-pressure torsion. *J. Mater. Sci. Technol.* 52 218–225. 10.1016/j.jmst.2020.01.065

[B289] Sikder P. Koju N. Ren Y. Phares T. Lin B. Bhaduri S. (2018). Development of single-phase silver-doped antibacterial CDHA coatings on Ti6Al4V with sustained release. *Surf. Coatings Technol.* 342 105–116. 10.1016/j.surfcoat.2018.02.100

[B290] Silva D. Sousa H. C. D. Gil M. H. Santos L. F. Moutinho G. M. Serro A. P. (2018). Antibacterial layer-by-layer coatings to control drug release from soft contact lenses material. *Int. J. Pharm.* 553 186–200. 10.1016/j.ijpharm.2018.10.041 30342082

[B291] Simchi A. Tamjid E. Pishbin F. Boccaccini A. R. (2011). Recent progress in inorganic and composite coatings with bactericidal capability for orthopaedic applications. *Nanomed. Nanotechnol. Biol. Med.* 7 22–39. 10.1016/j.nano.2010.10.005 21050895

[B292] Simi V. S. Rajendran N. (2017). Influence of tunable diameter on the electrochemical behavior and antibacterial activity of titania nanotube arrays for biomedical applications. *Mater. Charact.* 129 67–79. 10.1016/j.matchar.2017.04.019

[B293] Singh A. V. Vyas V. Patil R. Sharma V. Scopelliti P. E. Bongiorno G. (2011). Quantitative characterization of the influence of the nanoscale morphology of nanostructured surfaces on bacterial adhesion and biofilm formation. *PLoS One* 6:e25029. 10.1371/journal.pone.0025029 21966403 PMC3180288

[B294] Siritongsuk P. Hongsing N. Thammawithan S. Daduang S. Klaynongsruang S. Tuanyok A. (2016). Two-phase bactericidal mechanism of silver nanoparticles against Burkholderia Pseudomallei. *PLoS One* 11:e0168098. 10.1371/journal.pone.0168098 27977746 PMC5158019

[B295] Sivaprakash V. Narayanan R. (2020). Synthesis of TiO2 nanotubes via electrochemical anodization with different water content. *Mater. Today Proc.* 10.1016/j.matpr.2020.04.657

[B296] Skoric L. Sanz-Hernández D. Meng F. Donnelly C. Merino-Aceituno S. Fernández-Pacheco A. (2020). Layer-by-layer growth of complex-shaped three-dimensional nanostructures with focused electron beams. *Nano Lett.* 20 184–191. 10.1021/acs.nanolett.9b03565 31869235

[B297] Son I. H. Park J. H. Kwon S. Choi J. W. Rümmeli M. H. (2016). Graphene coating of silicon nanoparticles with CO2-enhanced chemical vapor deposition. *Small* 12 658–667. 10.1002/smll.201502880 26662621

[B298] Song J. Zheng M. Zhang B. Li Q. Wang F. Ma L. (2017). Fast Growth of Highly Ordered TiO2 Nanotube Arrays on Si Substrate under High-Field Anodization. *Nano Micro Lett.* 9:13. 10.1007/s40820-016-0114-4 30460310 PMC6223787

[B299] Spengler C. Nolle F. Mischo J. Faidt T. Grandthyll S. Thewes N. (2019). Strength of bacterial adhesion on nanostructured surfaces quantified by substrate morphometry. *Nanoscale* 11 19713–19722. 10.1039/c9nr04375f 31599281

[B300] Stratakis E. Bonse J. Heitz J. Siegel J. Tsibidis G. D. Skoulas E. (2020). Laser engineering of biomimetic surfaces. *Mater. Sci. Eng. R Rep.* 141:100562 10.1016/j.mser.2020.100562

[B301] Su H. L. Chou C. C. Hung D. J. Lin S. H. Pao I. C. Lin J. H. (2009). The disruption of bacterial membrane integrity through ROS generation induced by nanohybrids of silver and clay. *Biomaterials* 30 5979–5987. 10.1016/j.biomaterials.2009.07.030 19656561

[B302] Subramaniyan S. B. Megarajan S. Vijayakumar S. Mariappan M. Anbazhagan V. (2019). Evaluation of the toxicities of silver and silver sulfide nanoparticles against Gram-positive and Gram-negative bacteria. *IET Nanobiotechnol.* 13 326–331. 10.1049/iet-nbt.2018.5221 31053697 PMC8676110

[B303] Sun H. Choi D. Heo J. Jung S. Y. Hong J. (2020). Studies on the drug loading and release profiles of degradable chitosan-based multilayer films for anticancer treatment. *Cancers* 12:593. 10.3390/cancers12030593 32150885 PMC7140006

[B304] Surmeneva M. Lapanje A. Chudinova E. Ivanova A. Koptyug A. Loza K. (2019). Decreased bacterial colonization of additively manufactured Ti6Al4V metallic scaffolds with immobilized silver and calcium phosphate nanoparticles. *Appl. Surf. Sci.* 480 822–829. 10.1016/j.apsusc.2019.03.003

[B305] Sviridov A. P. Osminkina L. A. Kharin A. Y. Gongansky M. B. Kargina J. V. Kudryavtsev A. A. (2017). Cytotoxicity control of silicon nanoparticles by biopolymer coating and ultrasound irradiation for cancer theranostic applications. *Nanotechnology* 28:105102. 10.1088/1361-6528/aa5b7c 28177935

[B306] Swami N. Cui Z. Nair L. S. (2010). Titania nanotubes: novel nanostructures for improved osseointegration. *J. Heat Transfer* 133:034002 10.1115/1.4002465

[B307] Takahashi S. Chiba H. Kato T. Endo S. Hayashi T. Todoroki N. (2015). Oxygen reduction reaction activity and structural stability of Pt-Au nanoparticles prepared by arc-plasma deposition. *Phys. Chem. Chem. Phys.* 17 18638–18644. 10.1039/c5cp02048d 26118789

[B308] Tang S. Zheng J. (2018). Antibacterial activity of silver nanoparticles: structural effects. *Adv. Healthc. Mater.* 7:1701503. 10.1002/adhm.201701503 29808627

[B309] Taylor S. R. Sieradzki K. (2003). The development of a multi-functional aerospace coating: considerations in the use of nano-dimensioned materials. *Prog. Org. Coatings* 47 169–173. 10.1016/S0300-9440(03)00136-X

[B310] Thinakaran S. Loordhuswamy A. M. Venkateshwapuram Rengaswami G. D. (2020). Electrophoretic deposition of chitosan/nano silver embedded micro sphere on centrifugal spun fibrous matrices – A facile biofilm resistant biocompatible material. *Int. J. Biol. Macromol.* 148 68–78. 10.1016/j.ijbiomac.2020.01.086 31931057

[B311] Tian C. X. Wang Z. S. Zou C. W. Tang X. S. Xie X. Li S. Q. (2019). Ternary and quarternary TiBN and TiBCN nanocomposite coatings deposited by arc ion plating. *Surf. Coatings Technol.* 359 445–450. 10.1016/j.surfcoat.2018.12.081

[B312] Tranchant J. Angleraud B. Tessier P. Y. Besland M. P. Landesman J. P. Djouadi M. A. (2006). Residual stress control in MoCr thin films deposited by ionized magnetron sputtering. *Surf. Coatings Technol.* 200 6549–6553. 10.1016/j.surfcoat.2005.11.104

[B313] Tripathi B. N. Gaur J. P. (2004). Relationship between copper- and zinc-induced oxidative stress and proline accumulation in *Scenedesmus* sp. *Planta* 219 397–404. 10.1007/s00425-004-1237-2 15014994

[B314] Truong V. K. Geeganagamage N. M. Baulin V. A. Vongsvivut J. Tobin M. J. Luque P. (2017). The susceptibility of Staphylococcus aureus CIP 65.8 *and Pseudomonas aeruginosa ATCC* 9721 cells to the bactericidal action of nanostructured Calopteryx haemorrhoidalis damselfly wing surfaces. *Appl. Microbiol. Biotechnol.* 101 4683–4690. 10.1007/s00253-017-8205-9 28246886

[B315] Tsai C.-C. Teng H. (2004). Regulation of the physical characteristics of Titania Nanotube Aggregates Synthesized from Hydrothermal Treatment. *Chem. Mater.* 16 4352–4358. 10.1021/cm049643u

[B316] Tsai C.-C. Teng H. (2006). Structural Features of Nanotubes Synthesized from NaOH Treatment on TiO2 with Different Post-Treatments. *Chem. Mater.* 18 367–373. 10.1021/cm0518527

[B317] Tyurikova I. A. Alexandrov S. E. Tyurikov K. S. Kirilenko D. A. Speshilova A. B. Shakhmin A. L. (2020). Fast and Controllable Synthesis of Core-Shell Fe3O4-C Nanoparticles by Aerosol CVD. *ACS Omega* 5 8146–8150. 10.1021/acsomega.0c00392 32309724 PMC7161060

[B318] Umrao S. Jeon J. Jeon S. M. Choi Y. J. Lee S. (2017). A homogeneous atomic layer MoS2(1-: X)Se2 x alloy prepared by low-pressure chemical vapor deposition, and its properties. *Nanoscale* 9 594–603. 10.1039/c6nr07240b 27934991

[B319] Ur Rehman M. A. Bastan F. E. Nawaz Q. Goldmann W. H. Maqbool M. Virtanen S. (2018). Electrophoretic deposition of lawsone loaded bioactive glass (BG)/chitosan composite on polyetheretherketone (PEEK)/BG layers as antibacterial and bioactive coating. *J. Biomed. Mater. Res. Part A* 106 3111–3122. 10.1002/jbm.a.36506 30216664

[B320] Utke I. Hoffmann P. Melngailis J. (2008). Gas-assisted focused electron beam and ion beam processing and fabrication. *J. Vac. Sci. Technol. B Microelectron. Nanom. Struct.* 26:1197 10.1116/1.2955728

[B321] Velic A. Tesfamichael T. Li Z. Yarlagadda P. K. D. V. (2019). Parametric study on nanopattern bactericidal activity. *Procedia Manuf.* 30 514–521. 10.1016/j.promfg.2019.02.072

[B322] Viana M. Fonseca A. S. Querol X. López-Lilao A. Carpio P. Salmatonidis A. (2017). Workplace exposure and release of ultrafine particles during atmospheric plasma spraying in the ceramic industry. *Sci. Total Environ*. 599-600 2065–2073. 10.1016/j.scitotenv.2017.05.132 28558429

[B323] Vimbela G. V. Ngo S. M. Fraze C. Yang L. Stout D. A. (2017). Antibacterial properties and toxicity from metallic nanomaterials. *Int. J. Nanomed.* 12 3941–3965. 10.2147/IJN.S134526 28579779 PMC5449158

[B324] Vishnu J. Manivasagam V. Gopal V. Bartomeu Garcia C. Hameed P. Manivasagam G. (2019). Hydrothermal treatment of etched titanium: a potential surface nano-modification technique for enhanced biocompatibility. *Nanomed. Nanotechnol. Biol. Med.* 20:102016. 10.1016/j.nano.2019.102016 31158499

[B325] Vuchkov T. Evaristo M. Yaqub T. Bin, Polcar T. Cavaleiro A. (2020). Synthesis, microstructure and mechanical properties of W–S–C self-lubricant thin films deposited by magnetron sputtering. *Tribol. Int.* 150:106363 10.1016/j.triboint.2020.106363

[B326] Wang D. G. Xiao F. H. Li Y. Ming X. C. Zhai J. Q. Chen C. Z. (2020). Properties of HA-based composite films fabricated by pulsed laser deposition with an in-situ heat treatment. *Surf. Coatings Technol.* 394 10.1016/j.surfcoat.2020.125863

[B327] Wang L. Deng X. Li J. Liao X. Zhang G. Wang C. (2014). Hydrothermal synthesis of tetragonal BaTiO3 nanotube arrays with high dielectric performance. *J. Nanosci. Nanotechnol.* 14 4224–4228. 10.1166/jnn.2014.7782 24738375

[B328] Wang L. Lu W. Qin J. Zhang F. Zhang D. (2008). Microstructure and mechanical properties of cold-rolled TiNbTaZr biomedical β titanium alloy. *Mater. Sci. Eng. A* 490 421–426. 10.1016/j.msea.2008.03.003

[B329] Wang L. Wang C. Dunand D. C. (2015). Microstructure and Strength of NiTi-Nb Eutectic Braze Joining NiTi Wires. *Metall. Mater. Trans. A Phys. Metall. Mater. Sci.* 46 1433–1436. 10.1007/s11661-015-2781-z

[B330] Wang L. Wang C. Zhang L. C. Chen L. Lu W. Zhang D. (2016). Phase transformation and deformation behavior of NiTi-Nb eutectic joined NiTi wires. *Sci. Rep.* 6:23905. 10.1038/srep23905 27049025 PMC4822122

[B331] Wang L. Xie L. Shen P. Fan Q. Wang W. Wang K. (2019). Surface microstructure and mechanical properties of Ti-6Al-4V/Ag nanocomposite prepared by FSP. *Mater. Charact.* 153 175–183. 10.1016/j.matchar.2019.05.002

[B332] Wang L. Xie L. Zhang L. C. Chen L. Ding Z. Lv Y. (2018). Microstructure evolution and superelasticity of layer-like NiTiNb porous metal prepared by eutectic reaction. *Acta Mater.* 143 214–226. 10.1016/j.actamat.2017.10.021

[B333] Wang L. N. Jin M. Zheng Y. Guan Y. Lu X. Luo J. L. (2014). Nanotubular surface modification of metallic implants via electrochemical anodization technique. *Int. J. Nanomed.* 9 4421–4435. 10.2147/IJN.S65866 25258532 PMC4172084

[B334] Wang X. Yan L. Ye T. Cheng R. Tian J. Ma C. (2019). Osteogenic and antiseptic nanocoating by in situ chitosan regulated electrochemical deposition for promoting osseointegration. *Mater. Sci. Eng. C* 102 415–426. 10.1016/j.msec.2019.04.060 31147012

[B335] Wang Y. Liu X. Fan T. Tan Z. Zhou Z. He D. (2017). *In vitro* evaluation of hydroxyapatite coatings with (002) crystallographic texture deposited by micro-plasma spraying. *Mater. Sci. Eng. C* 75 596–601. 10.1016/j.msec.2017.02.119 28415504

[B336] Wareing N. Szymanski K. Akkaraju G. R. Loni A. Canham L. T. Gonzalez-Rodriguez R. (2017). *In vitro* gene delivery with large porous silicon nanoparticles fabricated using cost-effective, metal-assisted chemical etching. *Small* 13:1602739. 10.1002/smll.201602739 28084695

[B337] Wategaonkar S. B. Pawar R. P. Parale V. G. Nade D. P. Sargar B. M. Mane R. K. (2020). Synthesis of rutile TiO2 nanostructures by single step hydrothermal route and its characterization. *Mater. Today Proc.* 23 444–451. 10.1016/j.matpr.2020.02.065

[B338] Watson G. S. Green D. W. Schwarzkopf L. Li X. Cribb B. W. Myhra S. (2015). A gecko skin micro/nano structure - A low adhesion, superhydrophobic, anti-wetting, self-cleaning, biocompatible, antibacterial surface. *Acta Biomater.* 21 109–122. 10.1016/j.actbio.2015.03.007 25772496

[B339] Wei W. Liu Y. Yao X. Hang R. (2020). Na-Ti-O nanostructured film anodically grown on titanium surface have the potential to improve osteogenesis. *Surf. Coatings Technol.* 397 10.1016/j.surfcoat.2020.125907

[B340] Whitesides G. M. (2005). Nanoscience, Nanotechnology, and Chemistry. *Small* 1 172–179. 10.1002/smll.200400130 17193427

[B341] Winkler R. Lewis B. B. Fowlkes J. D. Rack P. D. Plank H. (2018). High-Fidelity 3D-nanoprinting via focused electron beams: growth fundamentals. *ACS Appl. Nano Mater.* 1 1014–1027. 10.1021/acsanm.8b00158

[B342] Wong C. L. Tan Y. N. Mohamed A. R. (2011). A review on the formation of Titania nanotube photocatalysts by hydrothermal treatment. *J. Environ. Manage.* 92 1669–1680. 10.1016/j.jenvman.2011.03.006 21450395

[B343] Wu J. Wang X. Zhu B. He Q. Ren F. Tong F. (2020). pH-sensitive magnetic drug delivery system via layer-by-layer self-assembly of CS/PEG and its controlled release of DOX. *J. Biomater. Sci. Polym. Ed.* 31 1057–1070. 10.1080/09205063.2020.1740963 32175824

[B344] Wu S. Zuber F. Maniura-Weber K. Brugger J. Ren Q. (2018). Nanostructured surface topographies have an effect on bactericidal activity. *J. Nanobiotechnol.* 16:20. 10.1186/s12951-018-0347-0 29490703 PMC5830064

[B345] Wu Y. Long Y. Li Q. L. Han S. Ma J. Yang Y. W. (2015). Layer-by-Layer (LBL) self-assembled biohybrid nanomaterials for efficient antibacterial applications. *ACS Appl. Mater. Interfaces* 7 17255–17263. 10.1021/acsami.5b04216 26192024

[B346] Xia K. Zhang L. Huang Y. Lu Z. (2015). Preparation of gold nanorods and their applications in photothermal therapy. *J. Nanosci. Nanotechnol.* 15 63–73. 10.1166/jnn.2015.9586 26328306

[B347] Xia L. Long Y. Li D. Huang L. Wang Y. Dai F. (2019). LBL deposition of chitosan and silk fibroin on nanofibers for improving physical and biological performance of patches. *Int. J. Biol. Macromol.* 130 348–356. 10.1016/j.ijbiomac.2019.02.147 30817968

[B348] Xia Y. (2008). Nanomaterials at work in biomedical research. *Nat. Mater.* 7 758–760. 10.1038/nmat2277 18813296

[B349] Xin H. W. Tian L. H. Pan J. De, He Q. Xu Z. (2000). Synthesis of aluminum nitride films by activated reactive ion plating with a cathodic arc source. *Surf. Coatings Technol.* 131 167–170. 10.1016/S0257-8972(00)00756-8

[B350] Xin Q. Shah H. Nawaz A. Xie W. Akram M. Z. Batool A. (2019). Antibacterial carbon-based nanomaterials. *Adv. Mater.* 31 1–15. 10.1002/adma.201804838 30379355

[B351] Xingfang H. U. Shuyin Q. I. N. Jingfen T. Miefemg H. (1988). Selectively absorbing black aluminium coatingdeposited by vacuum evaporation. *Solar. Energy Mater.* 17 207–215.

[B352] Xu J. Xu N. Zhou T. Xiao X. Gao B. Fu J. (2017). Polydopamine coatings embedded with silver nanoparticles on nanostructured titania for long-lasting antibacterial effect. *Surf. Coat. Technol.* 320 608–613.

[B353] Xu J. W. Yao K. Xu Z. K. (2019). Nanomaterials with a photothermal effect for antibacterial activities: an overview. *Nanoscale* 11 8680–8691. 10.1039/c9nr01833f 31012895

[B354] Xu W. Qi M. Li X. Liu X. Wang L. Yu W. (2019). TiO2 nanotubes modified with Au nanoparticles for visible-light enhanced antibacterial and anti-inflammatory capabilities. *J. Electroanal. Chem.* 842 66–73. 10.1016/j.jelechem.2019.04.062

[B355] Xue F. Liu J. Guo L. Zhang L. Li Q. (2015). Theoretical study on the bactericidal nature of nanopatterned surfaces. *J. Theor. Biol.* 385 1–7. 10.1016/j.jtbi.2015.08.011 26343860

[B356] Yang E. J. Kim S. Kim J. S. Choi I. H. (2012). Inflammasome formation and IL-1β release by human blood monocytes in response to silver nanoparticles. *Biomaterials* 33 6858–6867. 10.1016/j.biomaterials.2012.06.016 22770526

[B357] Yang J. Zhang M. Lan X. Weng X. Shu Q. Wang R. (2018). Controllable fabrication of non-close-packed colloidal nanoparticle arrays by ion beam etching. *Nanoscale Res. Lett.* 13:177. 10.1186/s11671-018-2586-2 29892834 PMC5995763

[B358] Yang T. Wang D. Liu X. (2019). Assembled gold nanorods for the photothermal killing of bacteria. *Colloids Surfaces B Biointerfaces* 173 833–841. 10.1016/j.colsurfb.2018.10.060 30551299

[B359] Yang Y. C. Chen C. C. Wang J. B. Wang Y. C. Lin F. H. (2017). Flame sprayed zinc doped hydroxyapatite coating with antibacterial and biocompatible properties. *Ceram. Int.* 43 S829–S835. 10.1016/j.ceramint.2017.05.318

[B360] Yang Z. Gu H. Sha G. Lu W. Yu W. Zhang W. (2018). TC4/Ag metal matrix nanocomposites modified by friction stir processing: surface characterization, antibacterial property, and cytotoxicity *in Vitro*. *ACS Appl. Mater. Interfaces* 10 41155–41166. 10.1021/acsami.8b16343 30403843

[B361] Yang Z. Ma C. Wang W. Zhang M. Hao X. Chen S. (2019). Fabrication of Cu2O-Ag nanocomposites with enhanced durability and bactericidal activity. *J. Colloid Interface Sci.* 557 156–167. 10.1016/j.jcis.2019.09.015 31520996

[B362] Yasuyuki M. Kunihiro K. Kurissery S. Kanavillil N. Sato Y. Kikuchi Y. (2010). Antibacterial properties of nine pure metals: a laboratory study using *Staphylococcus aureus* and *Escherichia coli*. *Biofouling* 26 851–858. 10.1080/08927014.2010.527000 20938849

[B363] Yildiz Z. K. Atilgan A. Atli A. Özel K. Altinkaya C. Yildiz A. (2019). Enhancement of efficiency of natural and organic dye sensitized solar cells using thin film TiO2 photoanodes fabricated by spin-coating. *J. Photochem. Photobiol. A Chem.* 368 23–29. 10.1016/j.jphotochem.2018.09.018

[B364] Yilmaz O. Yorgancioglu A. (2018). “Nanocoatings: preparation, properties, and biomedical applications,” in *Polyme Nanomaterials in Nanotherapeutics*, ed. C. Vasile (Amsterdam: Elsevier Inc), 299–331. 10.1016/B978-0-12-813932-5.00008-X

[B365] Yin Y. Li Y. Cai W. Sui J. (2019). One-step deposition of antibacterial Ag@Pdop hybrid films on an NiTi alloy. *RSC Adv.* 9 29263–29272. 10.1039/C9RA05764A PMC9071848 35528435

[B366] Yu L. Jin G. Ouyang L. Wang D. Qiao Y. Liu X. (2016). Antibacterial activity, osteogenic and angiogenic behaviors of copper-bearing titanium synthesized by PIII&D. *J. Mater. Chem. B* 4 1296–1309. 10.1039/C5TB02300A 32262985

[B367] Yuvaraj D. Kaushik R. Narasimha Rao K. (2010). Optical, field-emission, and antimicrobial properties of ZnO nanostructured films deposited at room temperature by activated reactive evaporation. *ACS Appl. Mater. Interfaces* 2 1019–1024. 10.1021/am900792k 20423121

[B368] Zeng J. Ji X. Ma Y. Zhang Z. Wang S. Ren Z. (2018). 3D graphene fibers grown by thermal chemical vapor deposition. *Adv. Mater.* 30:e1705380. 10.1002/adma.201705380 29423926

[B369] Zhang C. Ding Z. Xie L. Zhang L. C. Wu L. Fu Y. (2017). Electrochemical and *in vitro* behavior of the nanosized composites of Ti-6Al-4V and TiO 2 fabricated by friction stir process. *Appl. Surf. Sci.* 423 331–339. 10.1016/j.apsusc.2017.06.141

[B370] Zhang K. Zhu Y. Liu X. Cui Z. Xianjin Y. Yeung K. W. K. (2017). Sr/ZnO doped titania nanotube array: an effective surface system with excellent osteoinductivity and self-antibacterial activity. *Mater. Des.* 130 403–412. 10.1016/j.matdes.2017.05.085

[B371] Zhang Q. Wu Z. Xu Y. X. Wang Q. Chen L. Kim K. H. (2019a). Improving the mechanical and anti-wear properties of AlTiN coatings by the hybrid arc and sputtering deposition. *Surf. Coatings Technol.* 378:125022 10.1016/j.surfcoat.2019.125022

[B372] Zhang Q. Xu Y. Zhang T. Wu Z. Wang Q. (2018a). Tribological properties, oxidation resistance and turning performance of AlTiN/AlCrSiN multilayer coatings by arc ion plating. *Surf. Coatings Technol.* 356 1–10. 10.1016/j.surfcoat.2018.09.027

[B373] Zhang R. Liu X. Xiong Z. Huang Q. Yang X. Yan H. (2018b). Novel micro/nanostructured TiO2/ZnO coating with antibacterial capacity and cytocompatibility. *Ceram. Int.* 44 9711–9719. 10.1016/j.ceramint.2018.02.202

[B374] Zhang R. Liu X. Xiong Z. Huang Q. Yang X. Yan H. (2018c). The immunomodulatory effects of Zn-incorporated micro/nanostructured coating in inducing osteogenesis. *Artif. Cells Nanomed. Biotechnol.* 46 1123–1130. 10.1080/21691401.2018.1446442 29517404

[B375] Zhang S. Xing M. Li B. (2018d). Biomimetic layer-by-layer self-assembly of nanofilms, nanocoatings, and 3D scaffolds for tissue engineering. *Int. J. Mol. Sci.* 19:1641. 10.3390/ijms19061641 29865178 PMC6032323

[B376] Zhang W. J. Hong C. Y. Pan C. Y. (2019b). Polymerization-Induced Self-Assembly of Functionalized Block Copolymer Nanoparticles and Their Application in Drug Delivery. *Macromol. Rapid Commun.* 40:e1800279. 10.1002/marc.201800279 29968349

[B377] Zhang X. Li C. Yu Y. Lu X. Lv Y. Jiang D. (2019c). Characterization and property of bifunctional Zn-incorporated TiO2 micro-arc oxidation coatings: the influence of different Zn sources. *Ceram. Int.* 45 19747–19756. 10.1016/j.ceramint.2019.06.228

[B378] Zhang X. Zhang D. Peng Q. Lin J. Wen C. (2019d). Biocompatibility of nanoscale hydroxyapatite coating on TiO(2) nanotubes. *Materials* 12:1979. 10.3390/ma12121979 31226733 PMC6630346

[B379] Zhao M. Gong H. Ma M. Dong L. Huang M. Wan R. (2019). A comparative antibacterial activity and cytocompatibility for different top layers of TiN, Ag or TiN-Ag on nanoscale TiN/Ag multilayers. *Appl. Surf. Sci.* 473 334–342. 10.1016/j.apsusc.2018.12.159

[B380] Zhao Y. Xing Q. Janjanam J. He K. Long F. Low K. (2014). Facile electrochemical synthesis of antimicrobial TiO2 nanotube arrays. *Int. J. Nanomed.* 9 5177–5187. 10.2147/IJN.S65386 25429214 PMC4243507

[B381] Zhen W. An S. Wang W. Liu Y. Jia X. Wang C. (2019). Gram-scale fabrication of Bi@C nanoparticles through one-step hydrothermal method for dual-model imaging-guided NIR-II photothermal therapy. *Nanoscale* 11 9906–9911. 10.1039/c9nr01557d 31089657

[B382] Zhou J. Zhang X. Sun J. Dang Z. Li J. Li X. (2018). The effects of surface topography of nanostructure arrays on cell adhesion. *Phys. Chem. Chem. Phys.* 20 22946–22951. 10.1039/C8CP03538E 30155544

[B383] Zhou W. Jia Z. Xiong P. Yan J. Li M. Cheng Y. (2018). Novel pH-responsive tobramycin-embedded micelles in nanostructured multilayer-coatings of chitosan/heparin with efficient and sustained antibacterial properties. *Mater. Sci. Eng. C* 90 693–705. 10.1016/j.msec.2018.04.069 29853141

[B384] Zhu C. Lv Y. Qian C. Ding Z. Jiao T. Gu X. (2018). Microstructures, mechanical, and biological properties of a novel Ti-6V-4V/zinc surface nanocomposite prepared by friction stir processing. *Int. J. Nanomed.* 13 1881–1898. 10.2147/IJN.S154260 29636607 PMC5880573

[B385] Zhu C. Lv Y. Qian C. Qian H. Jiao T. Wang L. (2016). Proliferation and osteogenic differentiation of rat BMSCs on a novel Ti/SiC metal matrix nanocomposite modified by friction stir processing. *Sci. Rep.* 6:38875. 10.1038/srep38875 27958394 PMC5153627

[B386] Zhu M. Liu X. Tan L. Cui Z. Liang Y. Li Z. (2020). Photo-responsive chitosan/Ag/MoS2 for rapid bacteria-killing. *J. Hazard. Mater.* 383:121122. 10.1016/j.jhazmat.2019.121122 31518801

[B387] Zhu W. Q. Shao S. Y. Xu L. N. Chen W. Q. Yu X. Y. Tang K. M. (2019). Enhanced corrosion resistance of zinc-containing nanowires-modified titanium surface under exposure to oxidizing microenvironment. *J. Nanobiotechnol.* 17:55. 10.1186/s12951-019-0488-9 30992009 PMC6466780

[B388] Zhu Y. Dong M. Chang K. Li J. Wang L. (2019). Prolonged anti-bacterial action by sluggish release of Ag from TiSiN/Ag multilayer coating. *J. Alloys Compd.* 783 164–172. 10.1016/j.jallcom.2018.12.295

[B389] Zhu Y. Liu X. Yeung K. W. K. Chu P. K. Wu S. (2017). Biofunctionalization of carbon nanotubes/chitosan hybrids on Ti implants by atom layer deposited ZnO nanostructures. *Appl. Surf. Sci.* 400 14–23. 10.1016/j.apsusc.2016.12.158

[B390] Zimmerli W. Sendi P. (2017). Orthopaedic biofilm infections. *APMIS* 125 353–364. 10.1111/apm.12687 28407423

[B391] Zong M. Bai L. Liu Y. Wang X. Zhang X. Huang X. (2017). Antibacterial ability and angiogenic activity of Cu-Ti-O nanotube arrays. *Mater. Sci. Eng. C* 71 93–99. 10.1016/j.msec.2016.09.077 27987791

